# Scalable Thermoset Informatics Integrated with Autonomous Experimentation for On-Demand Discovery

**DOI:** 10.1021/acs.chemmater.6c01600

**Published:** 2026-10-09

**Authors:** Marcus R. Fratarcangeli, Ayush Jain, Farzad Gholami, Jiaqi Nie, Anagha Savit, H. Jerry Qi, Rampi Ramprasad

**Affiliations:** † School of Mechanical Engineering, 1372 Georgia Institute of Technology, Atlanta, Georgia 30332, United States; ‡ School of Materials Science and Engineering, Georgia Institute of Technology, Atlanta, Georgia 30332, United States; § School of Computational Science and Engineering, Georgia Institute of Technology, Atlanta, Georgia 30332, United States

## Abstract

As photopolymer additive manufacturing matures, there is an increasing need for on-demand, application-specific materials. A major bottleneck is the limited exploration of the vast design space for new thermoset polymer formulations, which must satisfy both material property requirements and processing constraints. This challenge arises from the combinatorial complexity of formulations and the high cost of experimental evaluation. In this work, we present an end-to-end pipeline that integrates a partial self-driving laboratory (p-SDL) with informatics-driven optimization to discover and validate thermoset acrylate formulations. The pipeline begins with a thermoset acrylate database that consolidates multiple data sources, including in-house experiments, molecular dynamics simulations, and literature data. These data sets are used to train single-task and multitask predictive models, which interface directly with the p-SDL to guide experiments and measure mechanical and processing-related properties. Each component is validated independently and then deployed together to optimize a target elastomeric material with over 90% reduction in human effort. The complete optimization required only ∼40 h of active experimental time, demonstrating rapid identification of formulations that satisfy multiple target properties without exhaustive screening. This work establishes a scalable framework for embedding multifidelity machine learning within laboratory workflows, enabling autonomous, data-driven thermoset formulation discovery.

## Graphical abstract

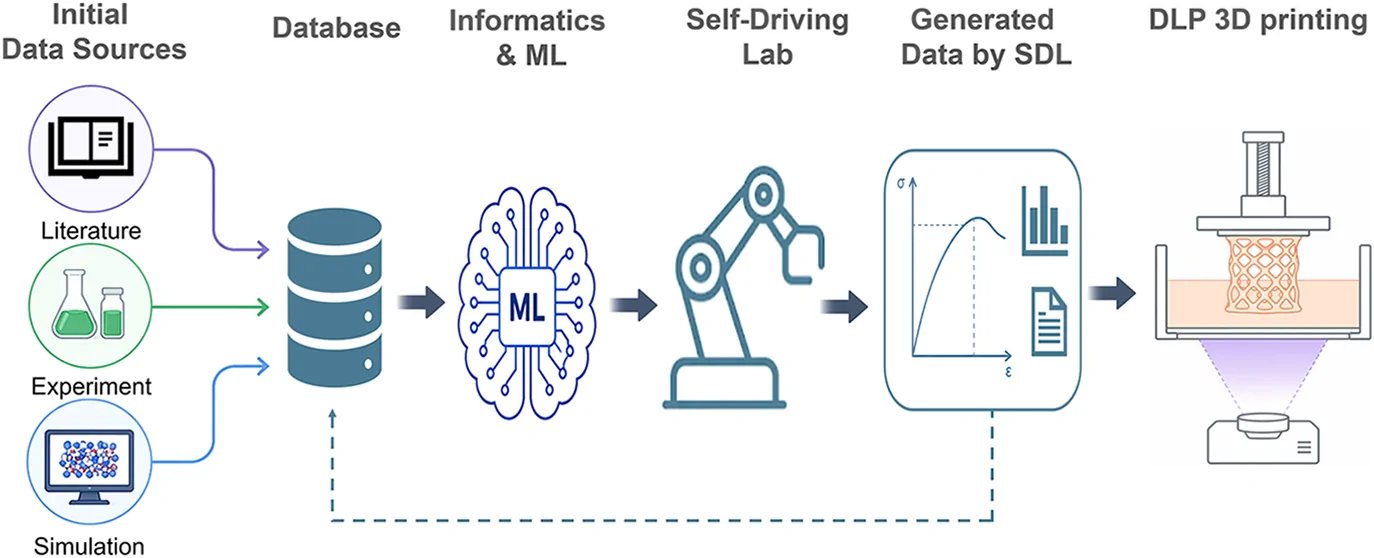

## 1 Introduction

Photocurable acrylates are a cornerstone of additive manufacturing (AM),^1^ particularly in vat photopolymerization techniques such as digital light processing (DLP), which enable the rapid fabrication of complex structures.^2,3^ The versatility of acrylate chemistry, which enables network formation from diverse monomers and cross-linkers, opens a vast, largely unexplored compositional space.^4,5^ This offers the potential to create bespoke materials with precisely tailored mechanical, thermomechanical, and processing properties on a per-need basis. However, the current selection of validated photopolymer resins for AM is narrow, forcing compromises for downstream production.^6^

Although machine learning (ML) offers a powerful route for exploring the vast design space of homopolymers,^7,8^ copolymers,^9^ and blends,^10^ thermosets and acrylates have not been explored at scale. The primary obstacle to expanding this material library is the experimental effort required and the processing parameters to control (i.e., UV light intensity, time of light exposure, and environmental temperature). Active learning helps reduce this burden by efficiently guiding experiments,^11^ but its impact is limited by experimental throughput. Automation through self-driving laboratories (SDLs) can overcome this challenge, enabling high-throughput formulation and characterization.^12–15^ Equally important is the integration of diverse data sources through multitask^16^ and multifidelity learning,^17^ which unifies experimental, simulated, and literature data. Together, these advances accelerate discovery and establish a scalable framework for data-driven materials innovation.

In this work, we present an end-to-end pipeline embedded directly into a partial self-driving laboratory (p-SDL) for the on-demand discovery of thermoset acrylate resins that target desired mechanical properties. As shown in Figure 1, our pipeline starts with data integration (Figure 1a) from the p-SDL, manual experiments (from our earlier work), molecular dynamics simulations,^18^ as well as leveraging a collection of databased literature experiments for thermoset polymers, *ThermosetDB* (Figure 1b). The data is then used in informatics tools to build models for predicting the properties of future thermoset formulation candidates (Figure 1c). Finally, we use the models for Bayesian and Gradient-based optimization (Figure 1d), which interface directly with the p-SDL. The p-SDL receives candidates and automates the testing of processing variables and mechanical properties (Figure 1e). The new data is uploaded back to *ThermosetDB*, and is used to retrain models for future optimization. We demonstrate the use of this framework to optimize an elastomeric material with up to 90% reduced human time in experimentation and formulation trials. The informatics pipeline, combined with diverse chemical data and connection to the p-SDL, creates a scalable and chemically flexible framework to accelerate the discovery of high-performance, photocurable acrylates for next-generation additive manufacturing.

**Figure 1:**
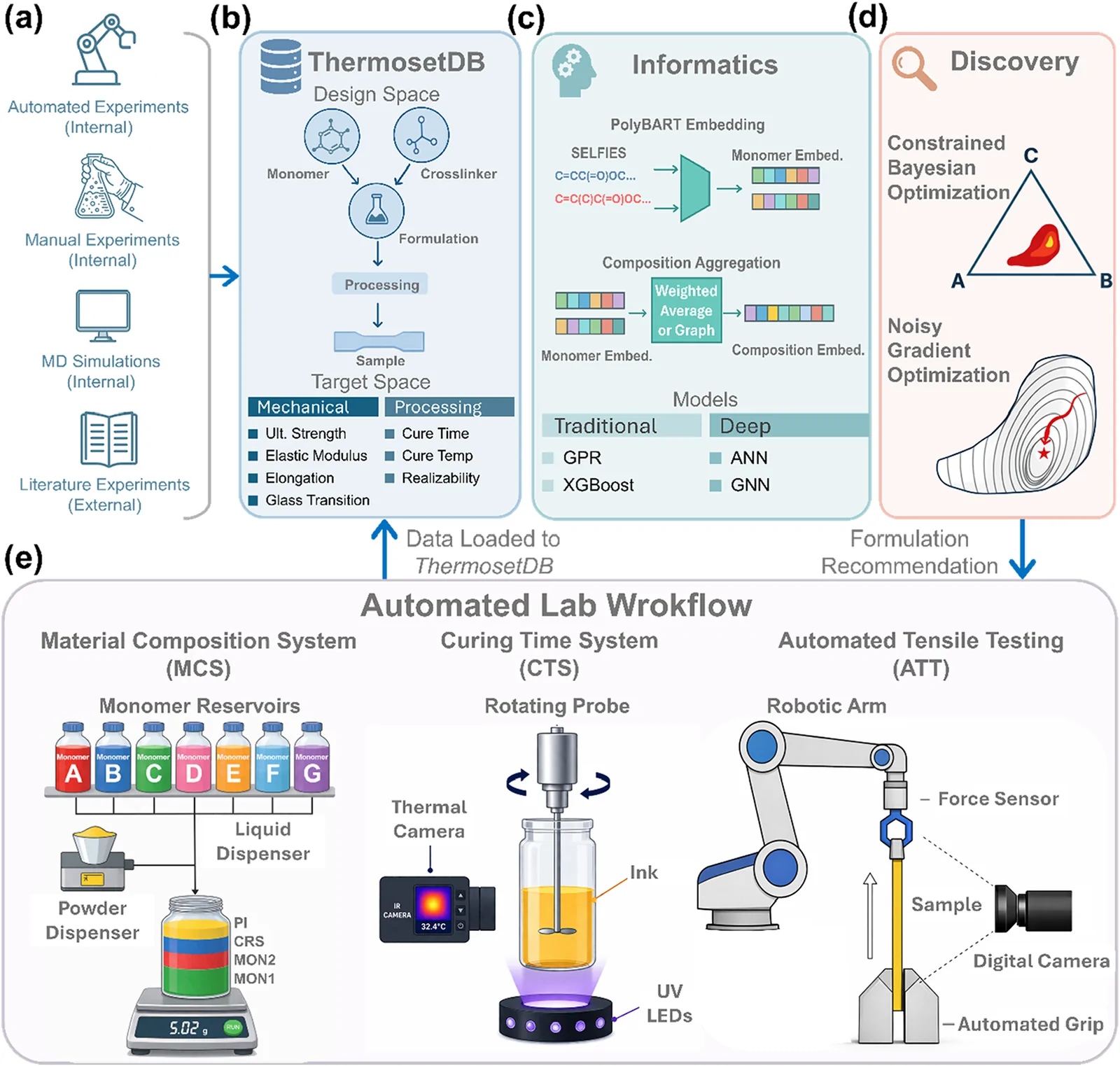
Overview of the end-to-end thermoset informatics pipeline. (a) Data from multiple sources, including partial self-driving laboratory (p-SDL) and manual experiments, molecular dynamics simulations, and literature experiments. (b) Setup of the *ThermosetDB* database schema to respect the multistep processing conditions that create thermosets. (c) Informatics of thermosets through the use of the polyBART embedding for monomers. These embeddings can be used in a mixture of shallow and deep models. (d) Acceleration of new formulations using Bayesian and Gradient optimization methods. (e) The p-SDL setup, which utilizes the recommendations from informatics. The p-SDL consists of 3 phases: formulation creation via the Material Composition System (MCS); in this part MON, CRS, and PI represent monomer, cross-linker, and photoinitiator, respectively, processing property determination through the Curing Time System (CTS), and mechanical property characterization via Automated Tensile Testing (ATT). The data collected is fed back into *ThermosetDB*.

## 2 Methodology

### 2.1 Experimental Methods

#### 2.1.1 Partial Self-Driving Lab Architecture

Our p-SDL system contains three primary components (Figure 1e): processing, characterization, and decision making.^19^ A detailed description of the p-SDL hardware layout is provided in Section 1 of the Supporting Information, with Figure S1a–c illustrating the complete experimental setup. First, the processing system is labeled as the Material Composition System (MCS), which automatically dispenses and mixes photopolymer resins. In addition, tensile bar fabrication is included in the processing component. Second, the characterization of the photopolymer resins is divided into two subsystems: the curing time system (CTS) and the automated tensile testing system (ATT). The CTS tests the curing time and temperature rise during curing. It then determines the resins’ realizability, defined as the curing time and temperature rise falling within certain thresholds for the resin to be considered usable for DLP printing. The ATT provides the modulus of elasticity (E), ultimate tensile strength (UTS), and elongation at break. Third, the decision-making component (Figure 1a–d) contains the active learning algorithm, which utilizes past data (in the first iteration) or the past data plus the CTS and ATT results (in subsequent iterations), builds a predictive model, and then provides several recommendations (four in this work) of resin compositions for the next experiments. The recommended compositions are then passed to MCS to start a new iteration.

Within this architecture, it is important to clarify the level of autonomy of the present SDL. Self-driving laboratories can operate at different levels of hardware and software autonomy, and complete automation of every experimental step is not required for a system to fall within the broader SDL framework. As described by Tom et al.,^12^ SDLs range from partially automated systems with human involvement to fully automated laboratories (six levels (0–5) on systems and four categories (0–3) on hardware and software, respectively). According to their classification, the platform presented in this work corresponds to a Level 4 SDL, with Category 2 autonomy in both hardware and software. In the present work, several experimental steps still require human intervention, and the platform should therefore be considered a partially automated SDL rather than a fully automated system. An important feature of the present platform is the integration of automated tensile testing into the SDL workflow. While mechanical characterization in previously reported SDLs has often relied on indentation-based measurements^20,21^ because of their ease of automation, tensile testing provides direct measurements of Young’s modulus, tensile strength, and elongation at break. To the best of our knowledge, the present work is among the first SDL implementations to integrate automated tensile testing for direct mechanical-property characterization.

##### 2.1.1.1 Material Composition System

The MCS comprises two primary components: the liquid dispenser for monomers and cross-linkers and the powder dispenser for photoinitiator­(Phenylbis­(2,4,6-trimethylbenzoyl)­phosphine oxide (PI819)). In this work, the MCS is limited to a total of five monomers (isodecyl acrylate (IDA), isobornyl acrylate (IBOA), 2-[[(butylamino)­carbonyl]­oxy]­ethyl acrylate (BCOE), benzyl methacrylate (BMA), and 2-hydroxyethyl acrylate (2-HEA)) and four cross-linkers (poly­(ethylene glycol) diacrylate at Mn of 250 and 700 (PEGDA-250, PEGDA-700), respectively, di­(ethylene glycol) dimethacrylate (DEGDMA), and trimethylolpropane ethoxylate triacrylate (ETPTA)) (chemical structure of the monomers and cross-linkers is presented in Figure S2) due to physical constraints. All chemicals are purchased from Sigma-Aldrich (St. Louis, MO). The formulation library consists of nine liquid reactive components, including five monomers and four cross-linkers. Each candidate formulation contains a maximum of three reactive components, with no more than two monomers and one cross-linker in a single formulation. The liquid dispenser consists of a series of peristaltic pumps powered by stepper motors.^22^ The step speed determines the amount of liquid dispensed. In addition, a proportional integral derivative control algorithm is implemented for accurate dispensing. During dispensing, the container is placed on a scale where the dispensing nozzles are overhead, and the scale reading is continuously monitored as feedback. The scale resolution is 0.003 g; compared to the total amount of dispensed liquid of 8 g, this is more than sufficient to minimize error in the early stages of the p-SDL. The photoinitiator powder dispenser similarly uses a stepper motor, which is connected to a pinion that turns a spur gear with a through hole on its face. There is a funnel containing the photoinitiator, aligned with the circular path of the spur gear hole. As the funnel exit aligns with the hole, the powder is dispensed and incentivized by a vibrating motor attached to the funnel. The revolution speed as well as the number of revolutions determine the amount of photoinitiator dispensed. Sampling the speed and mass spaces allows a curve fit between gear speed and dispensed mass, which can then be used to interpolate the required speed for any desired mass. It is worth mentioning that the photoinitiator is added directly to the low-viscosity monomer mixture and mixed with a magnetic stirrer at room temperature. At the concentrations used in this study (1 wt %), PI819 fully dissolves within several minutes of stirring, with no visible undissolved particles or sedimentation observed before printing. Besides the two dispensing systems, the MCS also has a 6-axis robotic arm (Epson C8-A901S, Seiko Epson Corporation, Nagano, Japan), with a custom gripper that moves the resin container between the liquid dispenser, powder dispenser, and mixer. Finally, a magnetic mixer is used to mix the resins after all the components are dispensed. Movie S1 demonstrates a complete loop of the MCS.

##### 2.1.1.2 Curing Time System

For a photopolymer resin to be realizable for 3D printing, its curing time should fall within a reasonable range. In addition, the temperature rise during curing cannot be too high. Although both curing time and temperature could be subjective, we use 10 s and 70 °C, respectively, as upper limit thresholds in this work. The choice of 10 s as the upper limit for curing time is guided by practical DLP processing constraints. In layer-by-layer photopolymerization, exposure times are typically kept within a few seconds to maintain reasonable printing speeds and limit heat accumulation from the exothermic reaction. Significantly longer exposures reduce throughput and can lead to overcuring and loss of spatial fidelity. Accordingly, 10 s is selected as a conservative upper bound representative of typical DLP operating conditions. It is worth noting that 70 °C is selected as the upper temperature limit for typical DLP 3D printing systems to prevent damage to the transparent film, which is commonly made of Teflon or fluorinated ethylene propylene (FEP). Our assumption of designing the CTS is based on two features of photocuring. First, the photopolymerization reaction is exothermic.^23^ Therefore, as the resin is cured, it is expected that the temperature will rise. Second, when the resin is cured from a liquid state to a solid state, its viscosity will increase, which can be detected through the change of a torque while stirring the resin. Based on these two assumptions, we designed the hardware comprising a robotic arm and a thermal camera. The robotic arm (the same model as the one in MCS) has a built-in torque sensor and an electromechanical servo gripper that places a probe into the photopolymer resin. Figure S3a,b shows far- and close-view of the CTS system. The data collection process begins with the robot’s wrist rotating slowly and turning the probe. At the same time, a thermal camera records the temperature of the resin. The UV LED lights are then turned on to begin the reaction. Both the torque and temperature are measured to determine the curing properties of each photopolymer resin through an automated data analysis process. Movie S2 provides a video demonstration of CTS operation. Typical temperature and torque evolution curves are presented in Figure S4a,b, respectively. To evaluate whether the temperature rise during CTS can induce thermal polymerization, three representative resin compositions from our data set are prepared including BCOE:DEGDAM at a weight ratio of 87.4:12.6, IDA:PEGDA250 at 75:25, and 2-HEA:PEGDA700 at 80:20. The resins are analyzed by DSC by heating from room temperature to 220 °C at a rate of 10 °C/min. As shown in Figure S5a, no exothermic event associated with polymerization is observed below 120 °C for any of the three systems. This temperature is substantially higher than the temperatures encountered during CTS, which typically remain near 55 °C and only rarely approach 80 °C. Fourier-transform infrared (FTIR) analysis is further used to examine whether exposure to elevated temperature produces detectable changes in the resin chemistry. For this experiment, 1 mL of each resin is heated in an 80 °C oven for 10 min, and the resulting FTIR spectra are compared with those of the corresponding as-prepared resins. As shown in Figure S5b, no significant spectral changes are observed after heating. To facilitate a more direct comparison, subtraction spectra obtained from the FTIR spectra before and after heating are presented in Figure S5c. These spectra further show no appreciable change in peak intensity following thermal exposure. The DSC and FTIR results indicate that the temperature increase generated during CTS does not induce significant thermal polymerization under the conditions investigated. Therefore, the polymerization detected by the CTS system is attributed predominantly to photoinitiated polymerization.

##### 2.1.1.3 Automated Tensile Testing System

The second system for characterization is for mechanical properties. The samples are first manually cured into dog-bone tensile bars in accordance with ASTM D638 Type IV for polymer materials (with a total length of 63.50 mm and a gauge length of 9.53 mm).^24^ Tensile specimens are prepared manually by dispensing the mixed resin into silicone molds, followed by UV curing at an intensity of 45 mW.cm^–2^ for 2 min. Because the uncured formulations investigated in this study have low viscosity and readily fill the molds, no degassing step is required. After curing and demolding, each specimen is visually inspected for obvious defects such as voids, warpage, or incomplete filling before mechanical testing. Specimens containing visible defects that can affect the measured mechanical response are excluded from testing. These samples are then placed on a rack for ATT testing. The ATT system consists of a robotic arm with an electromechanical servo gripper (the same one used in the CTS), which works with an in-house-built automated grip to secure the tensile bars from the bottom. The force is measured by the built-in force sensor. In addition, the robotic arm is equipped with a position tracking system, which continuously records its spatial position during the tensile test. During a tensile test, the robotic arm grabs one end of a sample from the rack, moves it, and inserts the other end into the automated grip, which then closes and secures the sample from the bottom. The robotic arm then moves vertically up by 90 mm at a displacement rate of 1 mm/s. The 90 mm travel distance is selected as the upper limit due to constraints in our robotic arm data acquisition system. Once the robotic arm reaches 90 mm of travel, the bottom grip opens. The robotic arm then moves to the sample damping area, opens its grip, and moves against a fixed brush to ensure the tested sample is brushed off. It then grabs another brush to clean the automated grip. This finishes one tensile test. During the test, the sample dimensions are measured using digital image correlation with two digital cameras.^25,26^ The ATT system also uses force and dimension to automatically obtain the stress–strain curve and extract mechanical properties, including *E*, UTS, and elongation at break. Movie S3 provides a video demonstration of ATT tests.

Since the p-SDL conducts tests autonomously, a concern to be addressed in such automated testing is verifying test integrity and detecting anomalies. In tensile testing, samples may contain defects and fail prematurely or break in an unexpected or undesirable manner. Therefore, an anomaly detection model consisting of two convolutional neural networks (CNNs) for the front and side views of the sample is developed to analyze and classify the breakage point of samples after a tensile test. A data set of 660 images are collected from the front and side cameras after tensile tests, where each frame is labeled as one of four classes: gauge break, low break, high break, sample stretched, with a split of 29.2%, 28.0%, 30.1%, and 12.7%, respectively. The gauge break implies the tensile bar break within the gauge region, high break means the sample breaks above the gauge region, low break means below the gauge region, and sample stretched means the sample does not break at all. The data set is split 90/10 for training and validation, and frames are cropped to 600 × 250 pixels and normalized before processing to reduce noise. Each CNN contained four convolutional layers with 7 × 7, 5 × 5, 3 × 3, and 3 × 3 kernels, respectively, and is trained using the *AdamW* optimizer and learning rate optimizer for 35 epochs. The final model uses the mean predictions from both the front and side CNNs to select the final class.

#### 2.1.2 Manual Characterization Techniques

The glass transition temperature (*T*_g_) of the samples was determined by dynamic mechanical analysis (DMA) using a Q800 instrument (TA Instruments, New Castle, DE). A temperature ramp of 5 °C/min is applied while oscillating the samples at 1 Hz with 0.1% strain. *T*_g_ is defined as the temperature corresponding to the peak in the tan δ signal. One DMA measurement is performed for each formulation due to the relatively large number of samples. To evaluate the repeatability of the DMA measurements, three replicate tests are conducted on a representative sample (PEGDA:GMA:AUD with a weight ratio of 70:25:5), and the corresponding tan δ curves are presented in Figure S6. The measured *T*_g_ values show less than 1 °C variation across the three replicates. Based on this repeatability, single measurements are used for the remaining formulations. The degree of conversion is determined by normalizing the FTIR peak intensity corresponding to the acrylate group at 809 *cm* ^*–1*^, measured using a Nicolet 6700 spectrometer (Thermo Fisher Scientific, Waltham, MA). Three FTIR measurements are performed for each sample to ensure reproducibility.

### 2.2 Informatics Methods

#### 2.2.1 Thermoset Fingerprinting

The state of the art in polymer fingerprinting is largely driven by tools such as Polymer Genome^27^ and a range of graph-^8^ or language-based models.^28^ These approaches are primarily designed for linear polymers and do not naturally extend to thermosets, where monomers can have multiple reaction sites, enabling the formation of cross-linked networks. To address this limitation, our representation scheme for thermoset polymers utilizes a weighted sum of numerical chemical fingerprints corresponding to the monomers that form the network. We use *polyBART*, an encoder-decoder based framework that has been trained on SELFIES representations of polymers and molecules,^29^ to create embeddings of monomers (Figure 1C). The most intuitive way to aggregate the monomer fingerprints to create the thermoset fingerprint *F* is through a composition-weighted average of the fingerprints of each constituent *f*_*i*_ as shown in eq 1,

1 $$F=\frac{1}{N}\sum_{i}^{N}m_{i}f_{i}$$

where *m*_*i*_ is the molar ratio of the specific composition.

However, the influence of different monomers is not determined solely by their molar ratios, but also by their functionality. For example, PEGDA, a cross-linker with two functional groups, may exert a greater influence on the physical properties of thermosets compared to a monomer such as glycidyl methacrylate (GMA), which has only one functional group. Thus, a fingerprinting method must account for the fact that each component depends on the others in the formulation. For this reason, we adopted a *reaction-graph-based* ^30^ approach to fingerprinting thermosets (a similar approach has been used by Goodall et al.^30^), explicitly incorporating reactivity and connectivity patterns into the representation to better learn the structural and functional complexity of cross-linked polymer networks from the composition ratios. Each molecular reaction system is represented as a graph *G = (V, E)*, where *V* denotes the set of nodes (atoms or molecular fragments) and *E* denotes the set of edges, which are the sets of reactions that occur. This is a fully connected graph. Each node *i* ∈ *V* is associated with an initial feature vector *x*_*i*_ ∈ *R* ^*d*_in_^ encoding atomic or fragment-level descriptors. The nodes are fully connected with loops. Node-representation *h*_*i*_ = *contat*([*x*_*i*_,*f*_*i*_]) where *f*_*i*_ is the mole fraction of the monomer. A multitask graph neural network (GNN) is used to learn a latent aggregation of the thermoset features and predict final properties (more details can be found in Supporting Information Section 2).

### 2.3 Molecular Dynamics Simulation Methods

Glass transition temperatures are calculated through cooling simulations using the CHARMM force field^31^ in GROMACS.^32^ The thermoset systems are created by starting with the monomers in a periodic simulation box at an initial density of between 250 to 350 kg/m^3^. 21-step equilibration is performed on the system, with a “mixing” step at 700 K to ensure that the monomers are evenly distributed. After equilibration, iterative curing cycles are conducted by finding nearby reactive acrylate functional groups, dragging them together, adding a bond to the topology, and then relaxing the system.^33^ This process is continued iteratively until the desired conversion rate is reached. The cured systems are energy minimized (10,000 steps, steepest descent), and subsequently preannealed under constant-temperature, constant-pressure (NPT) conditions (2,000 ps, 500 K, 10.0 bar, 0.001 ps time step) to facilitate gradual densification toward a physically realistic bulk density, particularly in cases where low cross-link density leads to initially expanded configurations requiring additional equilibration. Following a 21-step equilibration ending at 650 K, systematic cooling is performed from 650 to 75 K in 30 K decrements. At each temperature point, a two-stage protocol is employed: (1) NPT annealing with controlled linear cooling over 6,000 ps at 1.0 bar, and (2) isothermal NPT production at the target temperature for 5,000 ps. Timesteps are adaptively set to 0.001 ps for *T <* 250 K and 0.0005 ps for higher temperatures. This allows for stability during high vibrational activity but remains efficient when not necessary. System temperature and volume are monitored throughout each production run. The resulting volume vs temperature data is fitted using a hyperbolic function^34^ (more details can be found in Supporting Information Section 3) to determine the transition interval *T*_low_ to *T*_high_ where the *T*_g_ lies. Separate linear fits are performed on the low-temperature and high-temperature regions. Data points where *T* < *T*_low_ are fit to a linear function are considered the low temperature region and data points where *T* > *T*_high_ are fit to a linear function are considered a high temperature region. The *T*_g_ is determined as the intersection point of the two linear fits from the low-temperature and high-temperature regions. The simulations presented here are intended to capture high-level trends rather than provide a complete representation of all possible cross-linked network structures. Because only a limited number of cross-linked configurations are sampled and a classical force field is used, the quantitative accuracy may vary across different monomer chemistries. To ensure validity, we compute *R* ^2^ values for a general hyperbolic fit applied to the complete data set, a linear fit applied to the low-temperature segment, and a linear fit applied to the high-temperature segment.

### 2.4 Optimization Methods

#### 2.4.1 Bayesian Approach

The Bayesian optimization (BO) approach follows a scaled-up protocol of our previous work of machine-guided design in acrylates.^11^ A 7-dimensional design space is divided into increments of 5% weight ratios. We enumerate a candidate space of compositions with up to three types of monomers. The cross-linker ratios are capped to 50% so that the cross-linking would not overtake the properties of composition. The surrogate model is trained on previous trial data, and makes predictions on the candidate space. Unlike much of the theoretical work in multiobjective BO, materials design spaces are not smooth functions to estimate, but noisy functions with “islands” of different phases. The acquisition criteria are not always a maximization or minimization, and can include numerical targets, or ranges of satisfactory property values. To this end, a composite acquisition function is used to evaluate suitable candidates. Three distinct categories of design targets comprise this acquisition function, each having its own scoring given a candidate *x* and its posterior mean μ­(*x*) and standard deviation σ­(*x*)­

- **Maximize or Minimize**: 1D Expected Improvement (EI)^7,35^ formulation is used. Given the best observed value in the current data set, the acquisition is computed as eq 2
2 $$EI\;\left(x\right)=\{\begin{array}{ll} \alpha \;\left(\mu -f_{best}\right)\Phi \;\left(z\right)+\left(1-\alpha \right)\sigma \phi \;\left(z\right), & \mathrm{for}\;\mathrm{maximization}\\ \alpha \;\left(f_{best}-\mu \right)\Phi \;\left(z\right)+\left(1-\alpha \right)\sigma \phi \;\left(z\right), & \mathrm{for}\;\mathrm{minimization} \end{array}$$
  where $z=\frac{\left|\mu -f_{best}\right|}{\sigma }$ , Φ and ϕ are CDF and PDF of the standard normal distribution, and α balances exploitation and exploration.
- **Target**: The predictive distribution is transformed into a Gaussian function centered at the desired target value f*. For each candidate, samples are drawn as *f*(*x*) ∼ *N*(μ­(*x*), σ^2^(*x*)) and their squared deviation from the target (*f*(*x*) – f*)^2^ is computed. The distribution of this value can be framed as a minimization problem (the deviation decreases as *f*(*x*) gets closer to f*). Then, the EI is computed using the minimization form above. This formulation rewards candidates whose posteriors overlap with regions around f*.
- **Range**: This can be interpreted as adding constraints to the search by calculating the probability (*P*_range_(*x*)) of the posterior distribution falling into the desired range [*L*,*U*]. If the criteria have no lower bound (ex. a property only needs to be < *U*), then *L* = 0. If the criteria have no upper bound (ex. a property only needs to be > *L*), then *U* = *∞*. Formally, this is defined as eq 3,

3 $$P_{range}\;\left(x\right)=P\left(L\leq f\;\left(x\right)\leq U\right)=\Phi \left(\frac{U-\mu \;\left(x\right)}{\sigma \;\left(x\right)}\right)-\Phi \left(\frac{L-\mu \;\left(x\right)}{\sigma \;\left(x\right)}\right)$$

As a larger fraction of the posterior distribution lies within the desired range, the resulting score increases, favoring candidates with a higher likelihood of satisfying the imposed bounds. This probabilistic formulation naturally incorporates both the predicted mean μ­(*x*) and uncertainty σ­(*x*), enabling the selection of candidates that are both accurate and robust with respect to the target constraints. The range acquisition policy is target-directed rather than designed for global exploration of the full design space. Nevertheless, predicted uncertainty contributes directly to the probability of satisfying the specified bounds, allowing uncertain candidates near the target range to receive higher acquisition scores. Thus, the policy retains a degree of exploration while prioritizing identification of candidates within the desired property window. Because the Gaussian-process posterior is Gaussian and therefore unbounded, it can assign nonzero probability to negative values even for physically nonnegative target variables. This effect is negligible when the explored values remain sufficiently above zero, as in the present study, but may influence acquisition functions when predictions approach a physical lower bound. For such cases, positive-valued variables can be modeled after a logarithmic transformation or using other constrained formulations. An acquisition superscore can be given to candidates by taking an importance-weighted average of individual property scores, ∑_*i* = 1_^*N*_var._^ω_*i*_Α_*i*_(*x*), where Α_*i*_(*x*) is the objective acquisition score, and ω_*i*_ is a given weight (default is 1.0) for the *i* ^*th*^ target.

#### 2.4.2 Gradient Approach

The initial GPR-based Bayesian optimization operates on an enumerated formulation space with 5 wt % composition increments. After sufficient data are collected, the differentiable multitask GNN enables gradient-based optimization directly over the continuous composition simplex, providing finer formulation resolution than the original discrete search space. This transition is therefore motivated by search resolution and surrogate-model architecture rather than due to a limitation of Bayesian optimization for continuous variables. Gradient optimization is used to find compositions when a neural network architecture is trained on sufficient data from many sources, allowing a design space search that is not limited to enumeration. Although the formulation compositions serve as the input features during optimization, the multitask GNN used for the final optimization is trained using data from multiple sources, including the literature-derived data set. Thus, the literature data contribute to the learned composition–property relationships that guide the optimization, rather than being included as separate numerical input features. In this case, we use the multitask GNN and reaction graph approach. The optimization traverses the formulation simplex (a space where every point sums to 1) to find a composition of *n* components *x* = [*x*_1_, *x*_2_,..., *x*_*n*_] that minimizes a multiobjective loss function L as shown in eq 4,

4 $$\min_{x}L\left(x\right)=\sum_{k=1}^{K}\omega_{k}L_{k}\left(f_{k}\left(x\right),t_{k}\right)$$

where *f*_*k*_(*x*) denotes the GNN-predicted property *k*, *t*_*k*_ is target value or range, ω_*k*_ is a property-specific weight, and *K* is the number of properties under consideration. $L_{k}$ is the deviation of each goal from the target. From a starting point, which is a noised composition from the Bayesian step, we complete 10 forward passes through the GNN model using Monte Carlo dropout. L is calculated and the gradients of L with respect to the composition, $\frac{\partial L}{\partial x}$ , is calculated via autodifferentiation. A modified, simplex-aware Adam optimizer is then used to update *x*. This process is repeated multiple times until L converges. Through the iterations, sparse components are pruned dynamically from the graph representation, and we use randomized Dirichlet sampling at premature converences to escape local minima. Further details of this procedure, including relevant equations, are available in Supporting Information Section 4.

#### 2.4.3 Regret Calculation

Regret quantifies the deviation of the measured property values from their desired targets. A higher regret indicates that the sample is further away from the target. For a property *p* with observed value *x*_*p*_ and target *t*_*p*_, the normalized per-property regret is defined as eq 5

5 $$r_{p}=\{\begin{array}{ll} \frac{{\left(x_{p}-t_{p}\right)}^{2}}{max_{i}\;({x_{p,i}-t_{p})}^{2}}, & \mathrm{if}\;t_{p}\;\mathrm{is}\;a\;\mathrm{scaler}\;\mathrm{target}\\ \frac{{\max\;\left(0,t_{p}^{\mathrm{low}}-x_{p},x_{p}-t_{p}^{\mathrm{high}}\right)}^{2}}{max_{i}r_{p,i}}, & \mathrm{if}\;t_{p}\;\mathrm{is}\;a\;\mathrm{range}\;\mathrm{target}\;\left[t_{p}^{low},t_{p}^{high}\right] \end{array}$$

The total regret for a sample is computed as the summation over all goal properties, *R* = Σ_*p*_ *r*_*p*_.

## 3 Results and Discussion

### 3.1 Self-Driving Lab Performance

The p-SDL is used to conduct automated tests with the integrated active learning loop described above. Here, we use a palette of 9 monomers to encompass the chemical design space. The system is tested by runs over 18 generations. In each generation, four compositions are tested. For each composition, it is mixed once by MCS, three resins are tested by CTS, and eight samples are tested by ATT. To evaluate the p-SDL’s performance, we compare its time metrics of manual experimentation versus the actual human effort required to operate the p-SDL. Note that there are some tasks that needed human work in p-SDL, such as loading raw materials and containers, placing probes, preparing tensile samples, and placing the samples onto the rack.

Table 1 shows the overall efficiency metrics of the system, while Table 2 summarizes the acceleration achieved in the photopolymer data collection process per iteration. In Table 2, the time values correspond to the duration of running each system for a single iteration in our optimization loop, which consists of four compositions. Here, human time refers to the length of time during which a researcher must be actively present and working with the p-SDL for tasks mentioned above. The p-SDL reduces human time requirements by 91.3%, freeing more than 6 h of a typical workday for other research activities. Furthermore, the previously time-intensive task of manual data analysis for both characterization systems is now fully automated, achieving a 100% reduction in manual analysis time.

**Table 1:** Efficiency Metrics of the p-SDL

Metric	Value	Material Composition System	Curing Time System	Automated Tensile Testing
Time per sample (min)	*t*_*s*_	15	4	5
Samples per composition	*s*_*c*_	1	3	8
Samples per iteration	*s*_*i*_ = *s*_*c*_ × 4	4	12	32
Time per iteration (min)	*t*_*i*_ = *t*_*s*_ × *S*_*i*_	60	48	160

**Table 2:** Acceleration of Experimental Procedures via the p-SDL^a^

Metric	p-SDL run time (min)	Human time in p-SDL, t_human_(min)	Manual time, t_manual_(min)	Human time saved(%)
Material Composition Mixing	60	5	40	87.5
Curing Time Testing	48	10	60	83.3
Tensile Testing	160	20	180	88.9
Data Analysis	0	0	120	100
Total	268	35	400	91.3

a $h\mathrm{uman}\;\mathrm{time}\;\mathrm{saved}=(\frac{t_{manual}-t_{human}}{t_{manual}}\times 100)$

Figure 2 demonstrates the verification of the p-SDL systems. The MCS liquid dispensing system is tested by dispensing monomers at various target masses to verify the precision and accuracy of the system, while also determining the dispensing resolution, defined as the minimum mass increment that can be delivered reproducibly, as shown in Figure 2a. The results clearly show the system efficacy and minimization of error early in the p-SDL stages. The layer thickness of the resin in the CTS is about 3.2 mm, which is significantly higher than the typical layer thickness used in DLP printing. Therefore, we develop a simple model to map the tested photopolymer resins cure times to validate their use in DLP printing. The details of this model are provided in Section 5 of the Supporting Information. Figure 2b shows the relationship between the cure time measured in the CTS and DLP printer. Figure 2c compares tensile measurements obtained using the automated tensile testing (ATT) system with those from a commercial universal tensile testing machine (Criterion, MTS, Eden Prairie, MN). To evaluate the reliability of the ATT system over a broader range of mechanical behavior, two polymer samples with different stiffness levels are tested using both platforms. For Sample 1 (IBOA:2-HEA:AUD with ratios of 45:35:20), the specimens are stretched to 300% strain, and the two systems demonstrate close agreement in both the overall stress–strain response and the extracted mechanical properties (MTS: Young’s modulus = 34.3 ± 3.1 MPa, UTS = 6.2 ± 0.44 MPa; ATT: Young’s modulus = 35.6 ± 2.7 MPa, UTS = 6.3 ± 0.22 MPa). For Sample 2, a softer BCOE:DEGDMA formulation with a ratio of 87.4:12.6 is tested using both systems, and the resulting stress–strain curves are nearly identical (MTS: Young’s modulus = 2.10 ± 0.1 MPa, UTS = 1.65 ± 0.12 MPa; ATT: Young’s modulus = 2.12 ± 0.08 MPa, UTS = 1.53 ± 0.06 MPa). The agreement observed for both materials demonstrates the accuracy and reliability of the ATT system across samples with different stiffness and deformation behavior. This ATT system is developed primarily as a high-throughput characterization tool for materials discovery, where the resulting mechanical-property data are used as inputs for predictive modeling and optimization. Accordingly, the design priority is to achieve reproducible and sufficiently accurate measurements across a broad formulation space while maintaining automated throughput, rather than to replace conventional mechanical testing for detailed characterization of individual materials. The close agreement with the commercial MTS measurements indicates that the ATT system provides the level of accuracy required for this purpose.

**Figure 2:**
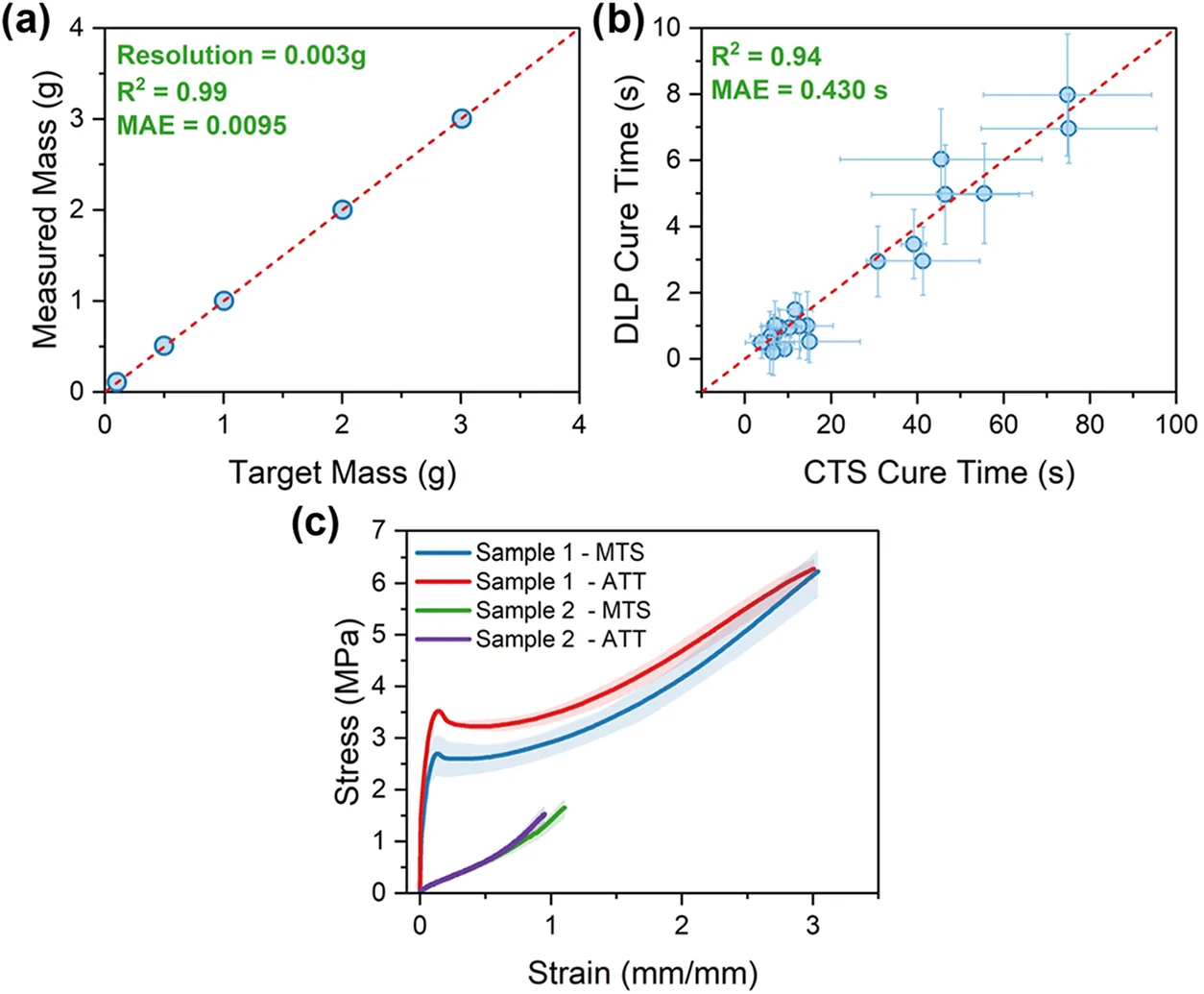
Accuracy of each part of the p-SDL. (a) Parity plot of the measured mass of the Material Composition System (MCS) vs the target mass. (b) Scaled parity plot of the measured curing time from the Curing Time System (CTS) vs the true cure time. (c) Comparison of the stress–strain curves for two different polymer samples with different stiffness ranges obtained from Automated Tensile Testing (ATT) and the MTS.

### 3.2 Acquired Data Sets

In this work, we collected data from in-house experiments (both manual and via p-SDL), the literature, and synthetically generated molecular dynamics simulations. For literature experiments, we constrain the collection to neat acrylate thermosets cured via UV radiation,^4,5,36–43^ with no reduction in the deviation of signals in the data set from dissimilar processing techniques. Figure 3 shows the summary of chemical and property distributions of literature and in-house experiments. In Figure 3a, in-house experiments are limited to a few main monomers accessible to our lab, and literature experiments include a diverse set of monomers. Supporting Information Section 6 contains the string representation SMILES (Simplified Molecular Input Line Entry System) of the acrylate monomers and cross-linkers considered across all data sets. In Figure 3b, we see that experiments are more clustered in the chemical space as the monomers used are limited to those available in our lab. With literature data sets, we can expand to more diverse chemical spaces while including some overlap between literature and in-house.

**Figure 3:**
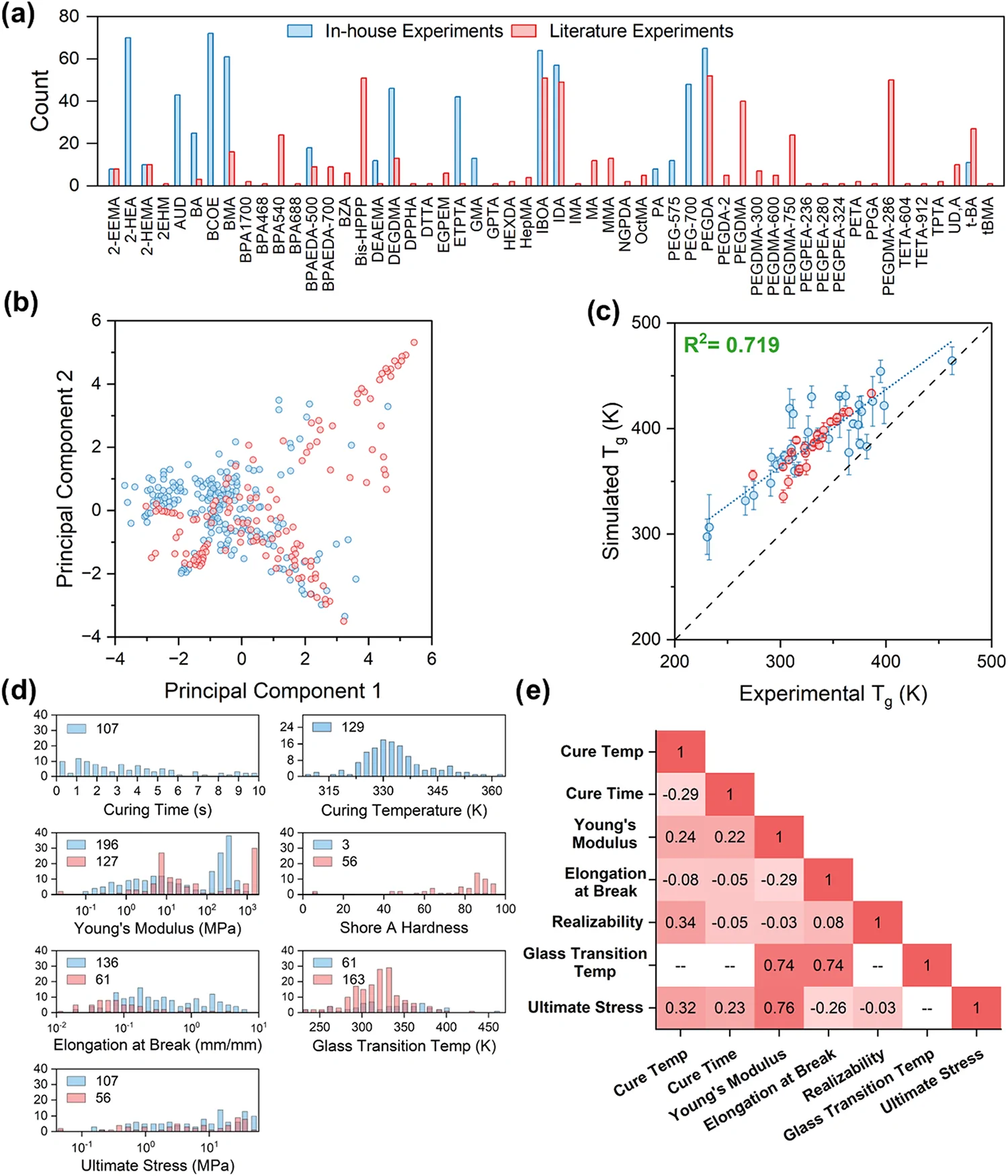
Data within ThermosetDB for both in-house (green) and literature experiments (orange). (a) Distribution of compositional occurrences in experimental data sets. (b) Visualization of the chemical-space based on compositional embeddings of the formulations after PCA reducing to 2 dimensions (46% total variance). (c) The glass transition temperature simulation alignment with in-house and literature experiments. (d) Property distributions from experimental data along with the in-house experiment and literature experiment counts. (e) Property correlations with experimental data in the in-house data points.

Classical molecular dynamics simulations have been used to simulate thermomechanical properties for linear polymers^44^ and thermosets,^45^ focusing on epoxies. In Figure 3c, we show simulated *T*_g_ values in comparison with both literature and in-house experiments. We conduct three trials per formulation, each with a different cross-linked configuration. Molecular dynamics simulations are performed only for values where the conversion percentage is reported. For any compositions with ranges of conversions, we linearly interpolate to estimate the *T*_g_ at intermediate conversions. The *T*_g_ calculated values from simulations are generally higher than those found experimentally. This is expected, as the time scales for molecular dynamics simulations are much shorter, so the polymer chains do not have time to relax and end up in a less ideal configuration, causing a higher observed transition point. Because this is a time scale problem, a correction factor can be estimated. The observed average difference between simulated and experimental *T*_g_ values is 58.3 K, corresponding to an estimated shift of ∼5.3 K per order of magnitude in cooling rate. The corrected simulation values have an *r* ^2^ = 0.719. Additionally, we see that using the conversion percentage can align the measurements from different data sources as it unifies the results of processing variations across literature.

Despite these high-level trends being simulated, there are shortcomings of the current simulation approach. The three cross-linked samples represent only a small subset of the possible network structures. Consequently, results based on only three trials may be influenced by nonrepresentative network configurations. Additionally, the use of a classical force field introduces limitations, as its accuracy may vary across monomers. The true potential of this approach lies in integrating simulations with experimental data sets within our informatics framework, which we explore in the subsequent section.

Figure 3d summarizes the collected property values for both in-house experiments and literature data. Our in-house experiments include a greater proportion of softer thermosets, with *E* < 1*GPa* and a high percentage of samples showing an elongation at break of >10%, whereas literature highlights stiffer materials. This distribution will change as materials are developed for increasingly diverse application needs and as the set of in-house experimental monomers expands. Figure 3e highlights correlations between collected property values, justifying further use of multitask modeling. The *T*_g_, *E*, elongation, UTS, and elongation at break hold strong correlations between each other. The resulting database provides a scalable backbone for the closed-loop optimization framework, enabling the training and benchmarking of machine learning methods, including the multitask models that drive optimization. By integrating in-house experimental data with literature sources, this approach also supports the flexible expansion of the accessible design space in future work.

### 3.3 Benchmarking of Machine Learning Models

As informatics at scale for acrylate thermosets is a gap in materials literature, it is important to benchmark the use of different fingerprinting and machine learning models. We compare several strategies, including a graph-based aggregation and a molar ratio weighted average aggregation. In the first approach, the GNN learns a fingerprint from monomer features and their molar ratios and predicts properties with a fully connected head. In the second approach, we use an artificial neural network (ANN), Gaussian process regression (GPR), and XGBoost (XGB) with the precombined polyBART fingerprints. We seek to validate the use of multiple data sources on model accuracy, similar to work conducted for homopolymers. We present model training over single-task (ST) data sets (one property at a time to build models from our in-house experiments), and multitask, multifidelity (MT_MF) data sets (combining all properties, in-house experiments and literature experiments in one model). All data set variations used in this study are retrieved from the curated thermoset database described above.

An important consideration is that the degree of curing, or conversion percentage from monomer to thermoset, greatly influences the properties.^18,23,29^ In theory, this conversion could be used as a predictive feature for property modeling. However, accurately measuring it requires infrared spectroscopy of both cured and uncured samples.^18^ This adds another layer of experimental effort, reducing the returns of adding this feature. Therefore, we train our models only on thermosets that have reached the conversion level at which polymerization stops. This value inherently depends on composition, so using the maximum attainable conversion provides a practical balance between model accuracy and experimental efficiency.

In Table 3, we show the model performance through the root mean squared error (RMSE) and linear correlation coefficient (*r* ^2^) between the predicted and ground truth values. Note that because E, UTS, and elongation at break span orders of magnitude, we compare them on the log scale. Generally, the GNN_MT_MF and ANN_MT_MF models have the highest performance, with both models exhibiting the first or second best RMSE and *r* ^2^ for every property except for cure time. The GNN-based model shows an advantage for scaling multitask thermoset informatics through the learned combination of the network components. The GNN_MT_MF model achieves consistently *r* ^2^ stronger performance for *T*_g_ from 0.107 to 0.564 and for cure temperature from 0.323 to 0.439 while maintaining competitive performance on other mechanical properties. Similar trends are seen for the ANN_MT_MF model, where the RMSE for *E* decreases from 1.3 to 0.51 log MPa when trained on external experimental data. Given that different properties arise from distinct underlying mechanisms captured by the fingerprint combinations, the optimal representation may vary. For example, cure time seems to be better mapped from a linear combination in the ANN approach, whereas *E* and cure temperature are better mapped from a learned aggregation via the GNN. Jointly training on in-house and literature data largely yields better performance for properties such as *E*, *T*_g_, ultimate stress, and cure temperature. A caveat of the MF approach is the potential introduction of conflicting signals from heterogeneous data sources. This is particularly evident for elongation at break, where the inclusion of external data increases the best RMSE from 0.33 and 0.34 (for GNN and ANN, respectively) to 0.34 and 0.36 log mm/mm, suggesting that data inconsistency can outweigh the benefits of increased sample size. Differences in model performance arise from both the representation used and the quality and quantity of the available data. Multitask and multifidelity models benefit from larger data sets and correlations among properties, while the GNN can additionally learn interactions among formulation components. However, heterogeneity in literature data can introduce additional noise, which may limit improvements for some properties. The 10-fold held-out data with the lowest RMSE and highest values are shown in Figure 4a–f. The receiver operating characteristic curve (Figure 4g) shows an area under the curve (AUC) of 0.88, indicating strong discrimination between realizable and unrealizable classes. These metrics are based on the current data set and are expected to improve as additional data is generated through continued operation of the automated platform.

**Figure 4:**
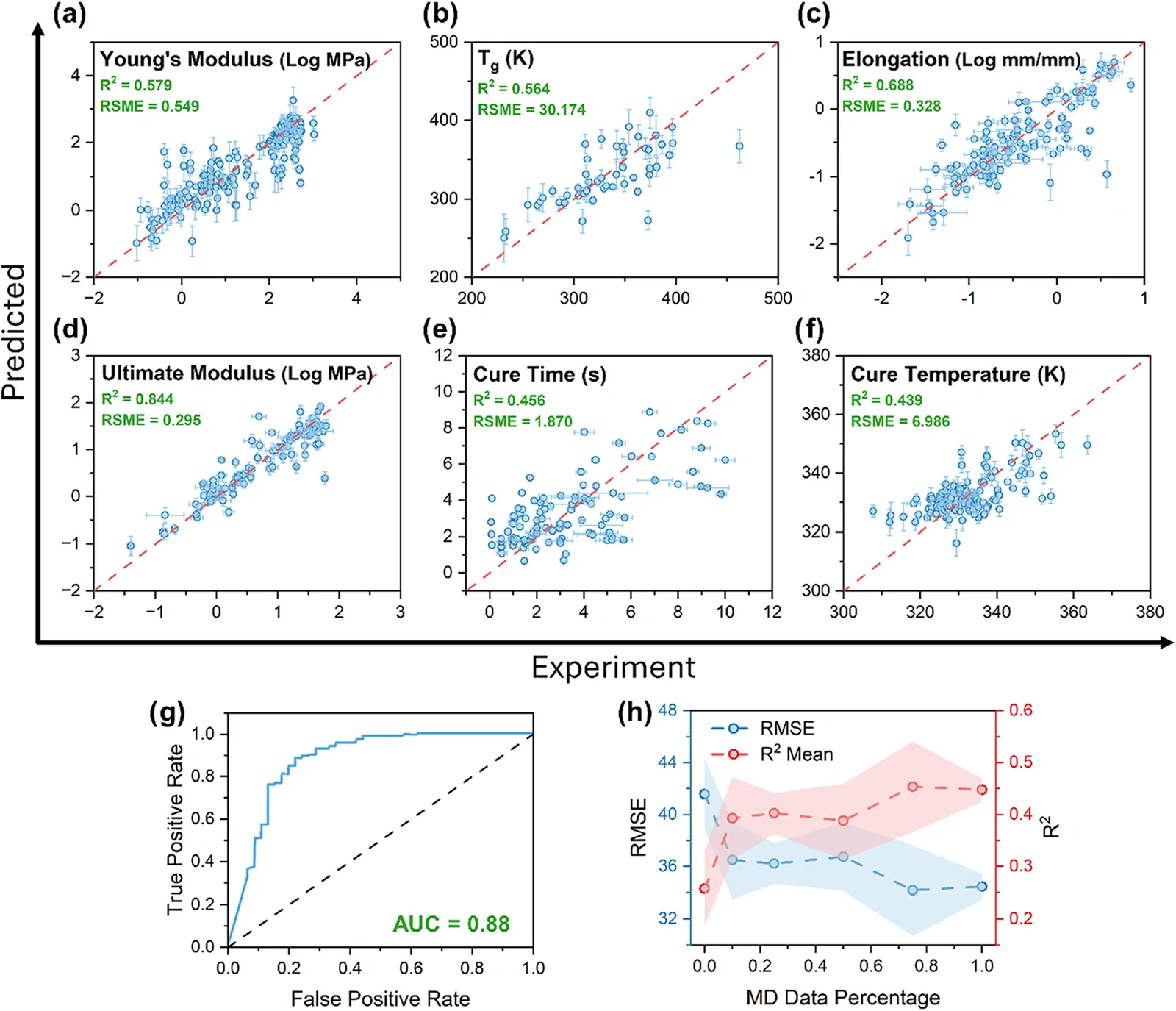
Results of selected best informatics benchmarks, aligned with the models high-lighted in 3. (a) *E* parity of GNN_MT_MF. (b) *T*_g_ parity of GNN_MT_MF. (c) max strain parity of GNN_ST. (d) UTS parity of ANN_MT_MF (e) Cure Time parity of GPR_MT_MF. (f) Cure Temperature parity of GNN_MT_MF. (g) Receiver Operating Characteristic of Realizability predictions from an ANN_ST. (h)­The RMSE and *R* ^2^ of the glass transition temperature predictions across different amounts of molecular dynamics data in the train set.

**Table 3:** Model Performance across Target Properties

	RMSE↓						*r* ^2^↑					
Model	E (log MPa)	*T*_g_(K)	Max Strain (logmm/mm)	Ult. Stress (MPa)	Cure Time (s)	Cure Temp (K)	E	*T*_g_	Max Strain	Ult. Stress	Cure Time	Cure Temp
GNN_MT_MF	0.55	30.1	0.34	0.30	2.26	6.98	0.76	0.56	0.67	0.83	0.20	0.44
ANN_MT_MF	0.56	34.4	0.36	0.29	2.02	7.65	0.74	0.43	0.61	0.84	0.01	0.33
GRN_MT_MF	0.62	31.2	0.44	0.43	2.26	7.02	0.69	0.53	0.43	0.66	0.24	0.43
XGB_MT_MF	0.73	34.5	0.49	0.47	2.16	7.40	0.56	0.43	0.27	0.60	0.17	0.38
GNN_ST	0.59	43.3	0.33	0.46	2.05	7.64	0.71	0.10	0.69	0.59	0.34	0.32
ANN_ST	1.32	59.7	0.34	0.31	2.06	7.91	–0.39	–0.7	0.67	0.81	0.34	0.28
GRN_ST	0.62	40.6	0.43	0.42	2.10	7.74	0.69	0.21	0.47	0.67	0	0.31
**XGB_ST**	1.08	63.7	0.37	0.46	1.93	7.16	0.05	–0.9	0.59	0.59	0.45	0.40

To assess the utility of molecular dynamics simulations for thermoset *T*_g_ with compositions that overlapped with the same compositions used in experiments, we also conducted an assessment of the prediction of *T*_g_ with variable amounts of molecular dynamics data. We conducted five 10-fold cross-validation splits with varied sampling of molecular dynamics data. In these low-data regimes, performance depends critically on which data points are sampled. In Figure 4h, we find that the distribution of RMSEs decreases from a mean of 41.8 at 0% and 36.53 at 10% of the molecular dynamics data to 34.47 K at 100%. These results demonstrate opportunities for further model improvement through strategic incorporation of molecular dynamics simulation data. Molecular dynamics data provide additional composition-dependent information for *T*_g_ prediction, but their benefit is also accompanied by uncertainties associated with force fields, cooling rates, and limited sampling of cross-linked network structures.

### 3.4 On-Demand Automated Discovery of Thermosets

The goal of this work is to discover new materials using the database and ML models built from thermoset database integrated with our p-SDL. Therefore, we implement the automated discovery framework that builds on the above-discussed pipeline to enable efficient optimization of formulations in a larger, flexible chemical design space and across multiple objectives. We explore a two-part optimization scheme. The first part is a BO approach using a GPR surrogate model to evaluate a discretized and constrained formulation space. The second part involves using the GNN_MT_MF model for gradient-based optimization, starting from a formulation chosen by the BO approach. This hierarchical approach allows the BO approach to constrain to high-priority areas after enumeration of the full design space, and the gradient optimization with the more accurate GNN enables continuous search over the local compositional simplex. Additionally, properties of materials over the full design space can be viewed as nonconvex, and direct optimization is difficult in this setting. By starting in an already constrained part of the manifold, we can more easily find a nonlocal optimum.

As mentioned above, our design space for the automated system contains five monomers: IDA, IBOA, BCOE, BMA, 2-HEA; and four cross-linkers: PEGDA-250, PEGDA-700, DEGDMA, and ETPTA. From these, our goal is to design a formulation with at most two monomers and one cross-linker. Adding more components beyond this yields diminishing returns for realizability and diversity in properties. To create an initial data set of samples and properties, we conduct an exploration of the design space for 18 iterations using diversity and uncertainty acquisition.^46^ Because the exploration phase has now established broad coverage of the design space, future optimization cycles can proceed directly without repeating this step. As the data set continues to grow over future optimization campaigns, model accuracy will likely increase as shown in Figure S8a,b in Supporting Information Section 7, enabling faster and more reliable optimization in subsequent iterations.

Following the exploration phase, we target an elastomeric material that balances strength and flexibility with a moderate stiffness of *E* = 1.2 MPa and an *UTS*/*E* ratio of >3, a high elongation at break *ε* > 200%, and low curing time of <5 s and temperature of <343 K. In the early iterations, we use the BO with a GPR surrogate. As more formulations are added to the data set and neural network–based models improve, we transition to the gradient-based optimization for higher precision and accuracy multitask learning. Figure 5a shows the regret of the best sample acquired from the desired target. Once the BO approach over an enumerated design space plateaus, applying gradient-based refinement to the selected candidate compositions enables the discovery of formulations with lower regret. In this particular case, the BO recommendation of 85.0% BCOE and 15.0% DEGDMA is refined into a final formulation of 87.4% BCOE and 12.6% DEGDMA using the gradient method, resulting in about 4 orders of magnitude drop in regret. Figure 5b shows highlights the specific ranges for each target. Here, we achieve final property values of *E* = 1.2 MPa, UTS = 3.7 MPa (UTS/*E* = 3.09), and elongation at break = 200.9%. In Figure 5c, the discovered formulation is printed as a slab and cyclically elongated to 150% strain. Lattice and gyroid structures are also fabricated and evaluated under cyclic compressive loading. As shown in Figure 5d in both the gyroid and cubic lattice geometries, the material exhibited minimal changes in hysteresis in the force vs displacement response after 10 compression cycles, indicating stable mechanical behavior. Movie S4 provides a video of compression testing of these lattice structures. The complete end-to-end optimization is accomplished within about 40 h of active experimental time (35.5 h p-SDL time +4.5 h human time), demonstrating a scalable framework for efficiently identifying formulations that satisfy stringent multiproperty targets without exhaustive experimental screening.

**Figure 5:**
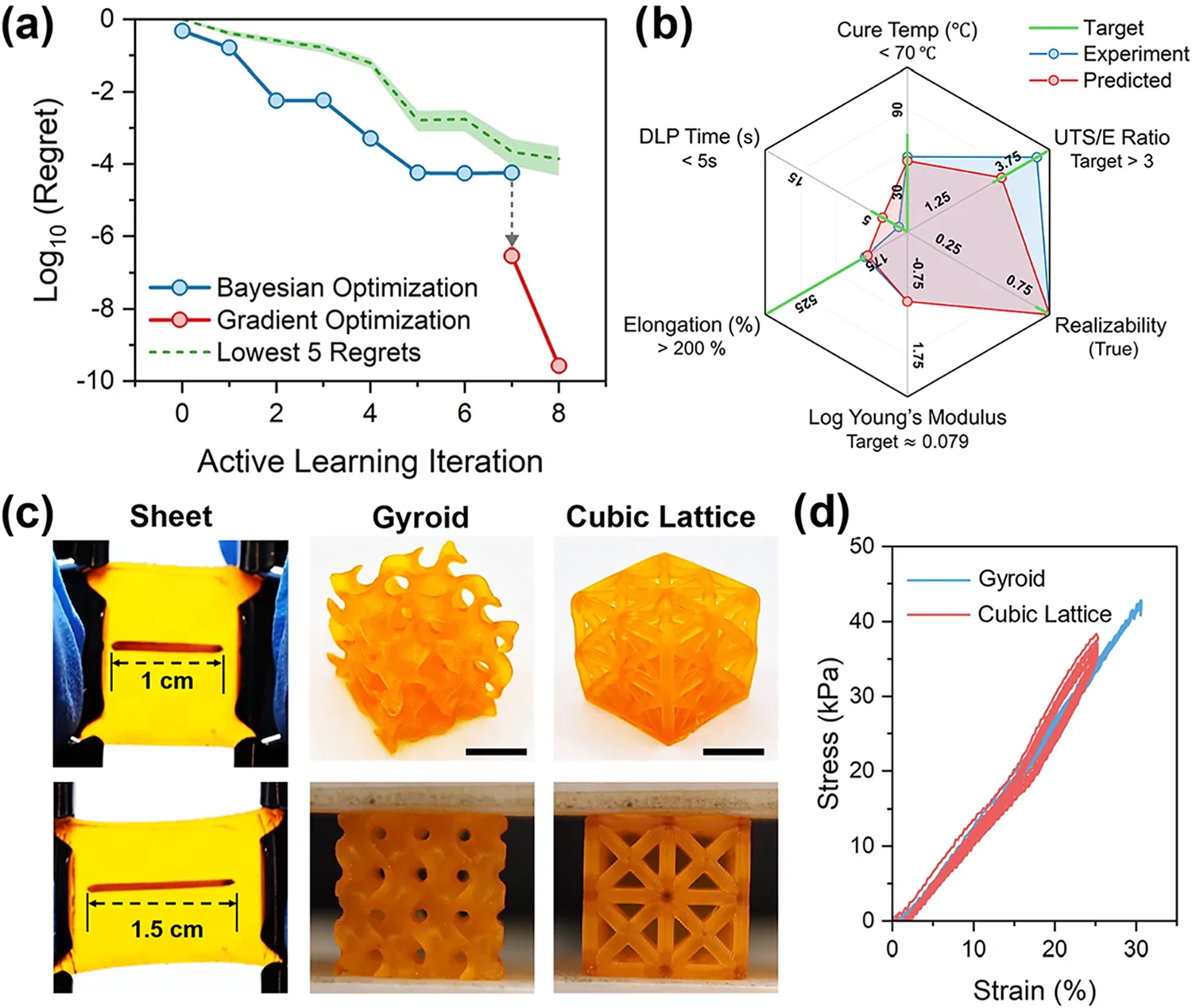
Results of automated materials discovery using the self-driving lab and the informatics protocols developed in this study. (a) The log-scaled regret of the best acquired sample after each iteration, first when using Bayesian Optimization and then further optimizing with gradient optimization when sufficient data is acquired. The distribution of the lowest 5 regrets in the data set is shown across the iterations. (b) A radar plot showing the properties of the optimized material composition. (c) Demonstrated printability and elastomeric responses of a thin sheet (left), gyroid (center), and a cubic lattice (right) structures (scale bars are 1 cm). (d) A demonstration of the cyclic compressive response is shown for the gyroid (3.0 mm displacement) and cubic lattice (2.5 mm displacement) structures.

## 4 Conclusion

We present an integrated framework for informatics-driven and automated discovery of thermoset acrylates by coupling machine learning–based surrogate models with an p-SDL for formulation and characterization workflows in a closed-loop system. The p-SDL accelerates data generation and enables iterative optimization, achieving more than a 90% reduction in human effort per discovery cycle while reducing the overall timeline from months to days. By prioritizing experiments through model-guided selection, the framework also minimizes material waste by reducing the total number of required experiments. Automated characterization also enables high-throughput experimentation, further enhancing the efficiency of the discovery process. Another key component of the approach is the integration of diverse data sources through multitask and multifidelity learning, combining simulations, literature data, and in-house experiments to improve model generalization and predictive accuracy for downstream optimization. We further demonstrate that simulated glass transition temperatures correlate with experimental values across varying chemistries and conversion rates, highlighting the potential of incorporating such descriptors into the active learning loop as additional multifidelity signals. Importantly, the end-to-end optimization is completed in ∼40 h of active experimental time (35.5 h p-SDL time and 4.5 h human time) to identify formulations meeting multiple target properties, highlighting the rapid convergence of the framework. This markedly shortens the time typically needed to discover materials with balanced mechanical performance and processing requirements. The framework represents an important step toward reducing reliance on traditional trial-and-error approaches in 3D printing material development. More broadly, the convergence of informatics, automation, and multisource learning establishes a scalable pathway toward accelerated and increasingly autonomous materials discovery for additive manufacturing.

## Supplementary Material











## Data Availability

The experimental and computational data supporting the findings of this study are available from the corresponding author upon reasonable request.

## Notes

A complete loop of the MCS (MP4)
A video demonstration of CTS operation (MP4)
A video demonstration of ATT tests (MP4)
A video of compression testing of these lattice structures (MP4)
Chemical structure of the monomers and crosslinkers used in MCS.; (a) entire p-SDL in lab space, (b) top view of the p-SDL mounted to table layout. and (c) close-up view of the ATT system (PDF)
