# Supplementary material for: Scalable Thermoset Informatics Integrated with Autonomous Experimentation for On-Demand Discovery
Source: Chem Mater. 2026 Oct 9;38(20):10562–75. doi: 10.1021/acs.chemmater.6c01600 (PMC13718556; doi:10.1021/acs.chemmater.6c01600)
Supplement: Supplementary file 1 [file cm6c01600_si_005.pdf]

Supplementary information for:

Scalable Thermoset Informatics Integrated with Autonomous Experimentation for On-Demand Discovery

Marcus R. Fratarcangeli,[1†] Ayush Jain,[2,3†] Farzad Gholami,[1,2†] Jiaqi Nie,[1] Anagha Savit,[2,3] H. Jerry Qi,[1] and Rampi Ramprasad[2*]

[1]School of Mechanical Engineering, Georgia Institute of Technology, Atlanta, GA 30332, United States

[2]School of Materials Science and Engineering, Georgia Institute of Technology, Atlanta, GA 30332, United States

[3]School of Computational Science and Engineering, Georgia Institute of Technology, Atlanta, GA 30332, United States

[†]Equal Contribution First Authors

*Corresponding authors, HJQ, qih@me.gatech.edu; RR, rampi.ramprasad@mse.gatech.edu

1 Partial Self-Driving Laboratory

The partial self-driving laboratory (p-SDL) resides on a custom-made modular table (Vention, Montreal, QC, Canada) that allows for hardware mounting anywhere on the surface. **Figure S1a**shows a depiction of the p-SDL table in the lab space and **Figure S1b** is the top view, showing the real arrangement of different components labeled, and **Figure S1c** is top view of automated tensile testing (ATT) system. The two robots are mounted at either end of the table and face the center, providing them with the largest work area possible. The right robot (Robot 1) manages the materials composition system (MCS) and is surrounded by other components: the container dispenser, the cork dispenser, the liquid dispensing system, the powder dispensing system, and the magnetic mixer. The left robot (Robot 2) is responsible for the characterization systems. In front of it is the automated grip and cameras, then the cleaning stations for the grippers, and to the right is the curing time system (CTS). Having the systems mounted to the same table allows them to potentially work together in future work. Additions can easily be made to the table to support an additional robot or subsystem to facilitate a completely autonomous p-SDL.

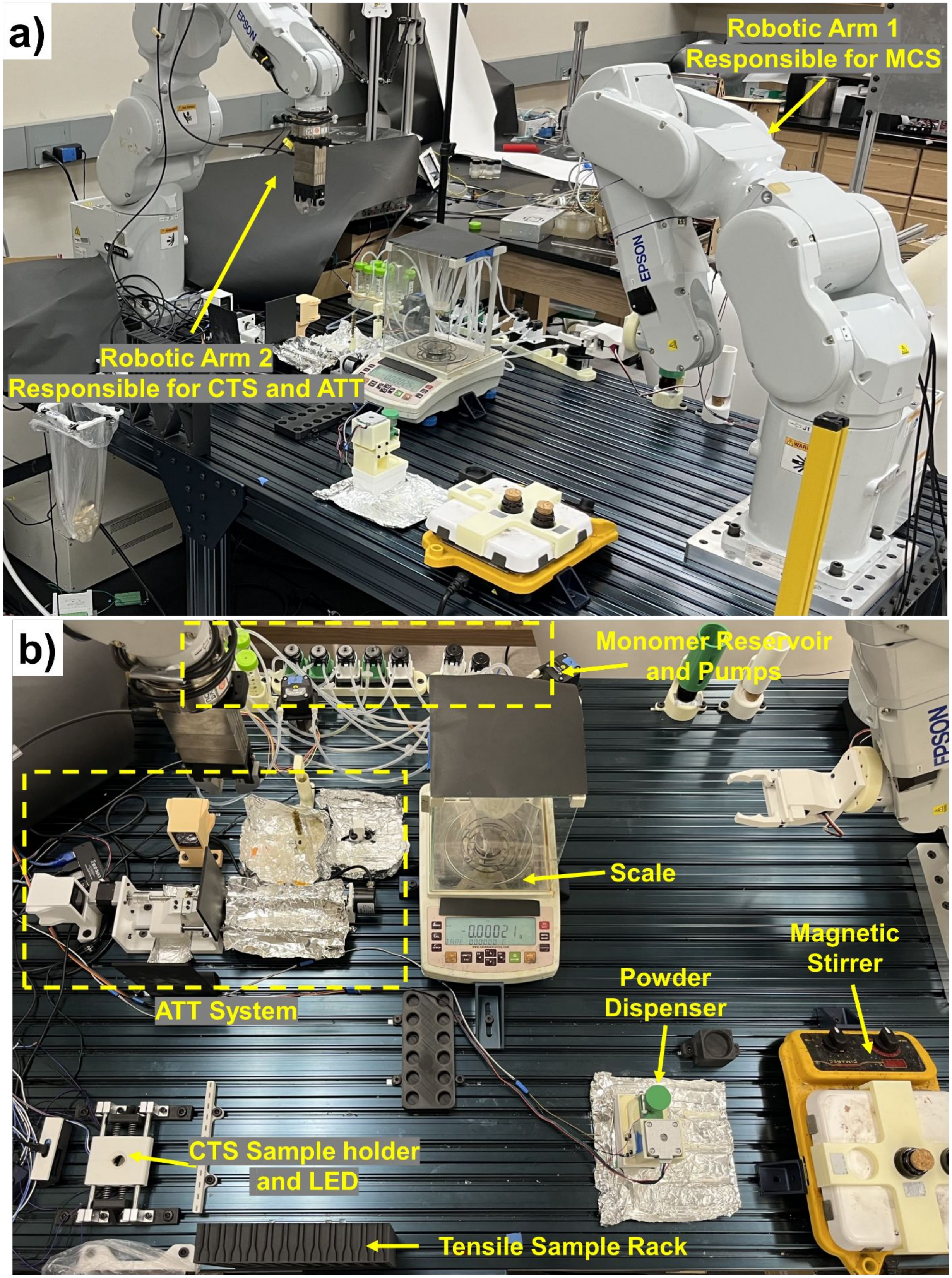
a)
Robotic Arm 1
Responsible for MCS
Robotic Arm 2
Responsible for CTS and ATT
b)
Monomer Reservoir
and Pumps
Scale
ATT System
Magnetic
Stirrer
Powder
Dispenser
CTS Sample holder
and LED
Tensile Sample Rack

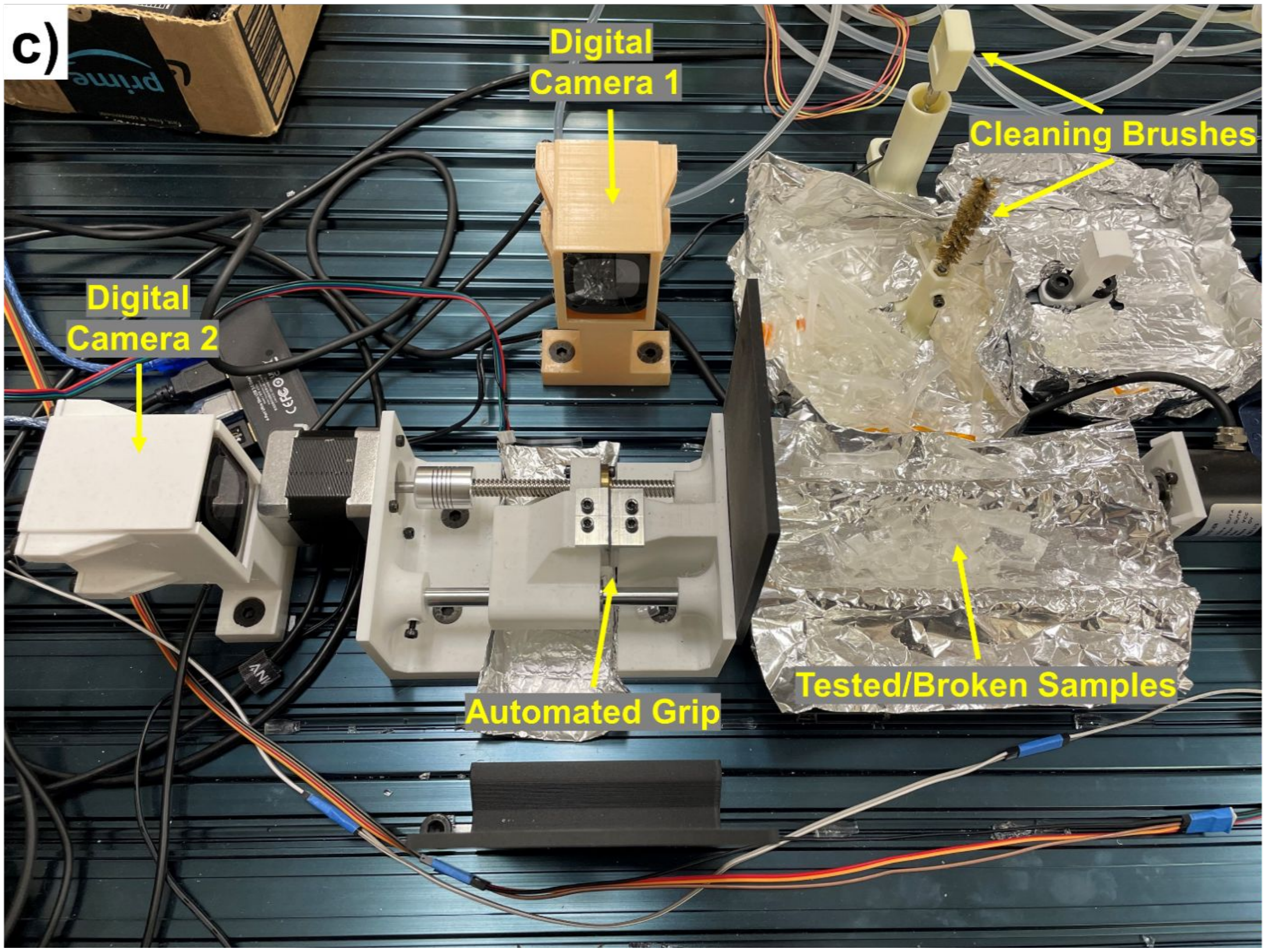

Figure S1. a) Entire p-SDL in lab space, b) top view of the p-SDL mounted to table layout. and c) close-up view of the ATT system.

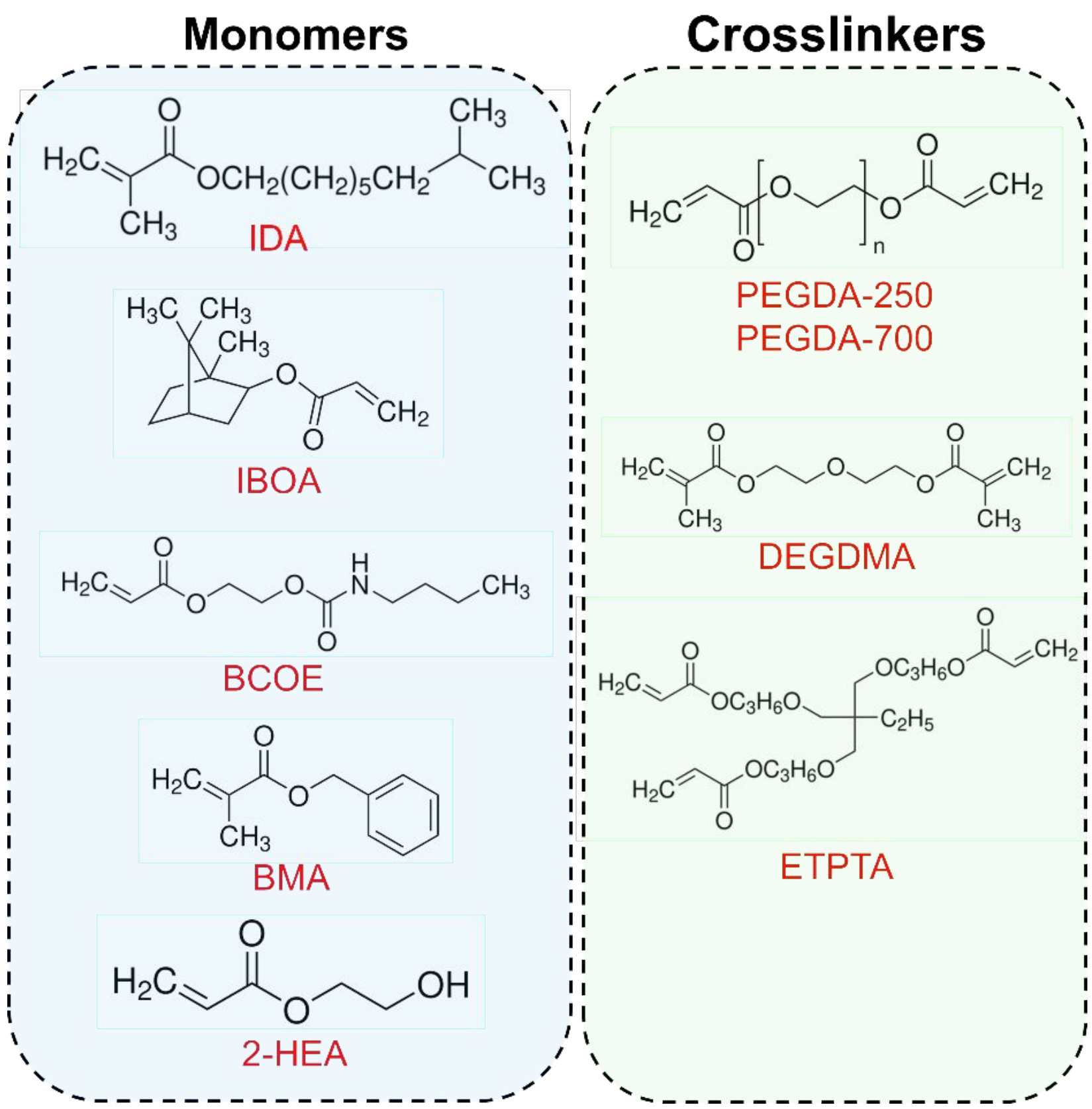

Figure S2. Chemical structure of the monomers and crosslinkers used in MCS.

The primary CTS hardware is shown in **Figure S3**, notably the robotic arm with force sensor and gripper, and the ink container/probe tools. The raw data collected from the CTS system are temperature and torque values. An example temperature curve after a single test of a photopolymer ink is demonstrated by **Figure S4a**. The UV light turns on after 30 seconds, and the ink temperature drastically rises as the heat is generated from bonds forming. The temperature then reaches its peak value as it solidifies and slowly begins cooling. The torque acting on the force sensor is measured simultaneously. Each point in **Figure S4b** represents the normalized amplitude of torque values, given that the gripper is oscillating the probe in the ink. The parameters shown on the plot are relevant to the fitted sigmoidal curve:

$$f = \frac{A}{1 + \exp\left(\frac{x-h}{s}\right)} + C \tag{S1}$$

where A is the parameter for the height of the top line relative to the bottom line, C is for the height of the bottom line, h represents the inflection point, and s is the distance between the top and bottom lines along the abscissa.

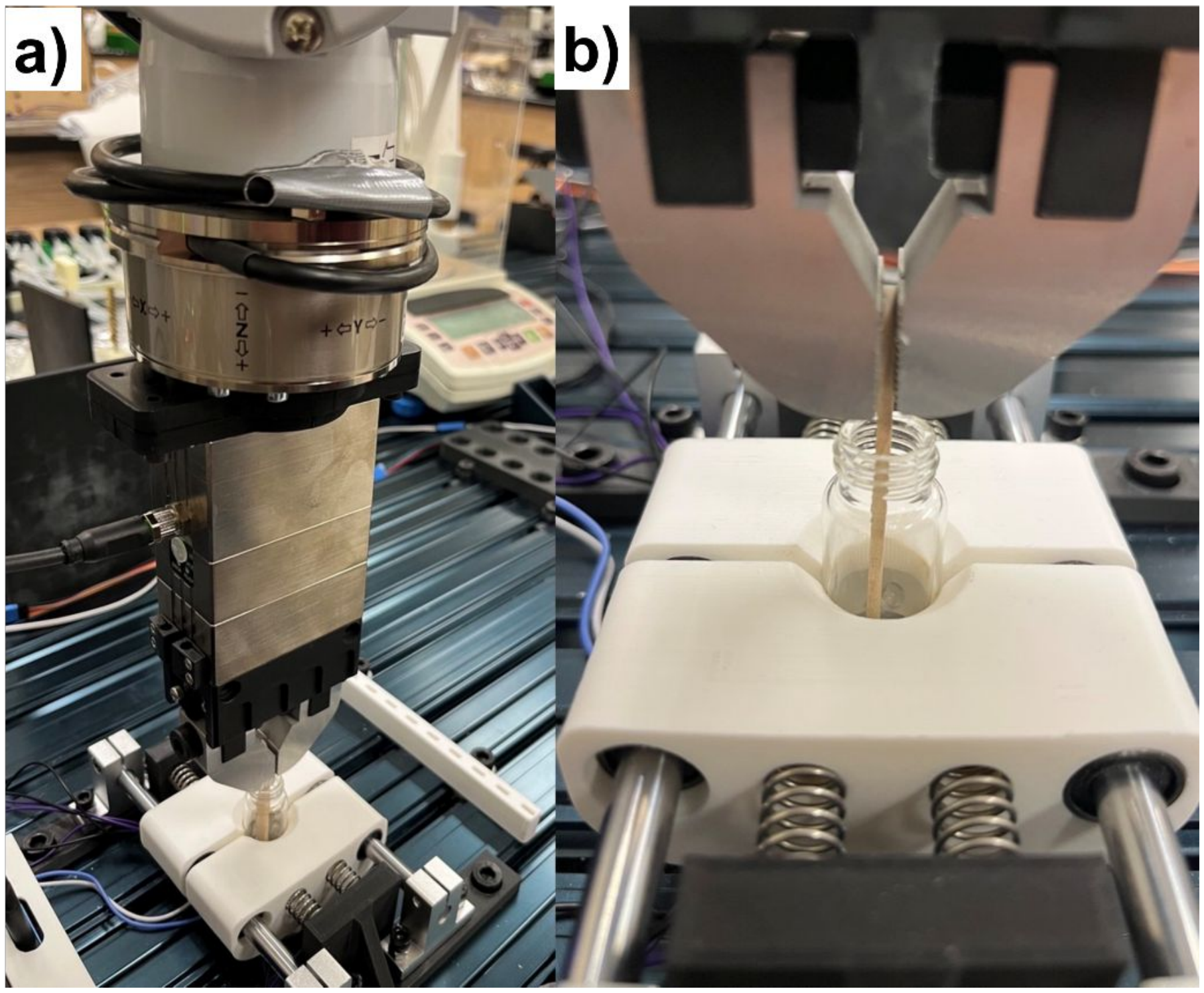

Figure S3. a) Far and b) close view of CTS hardware

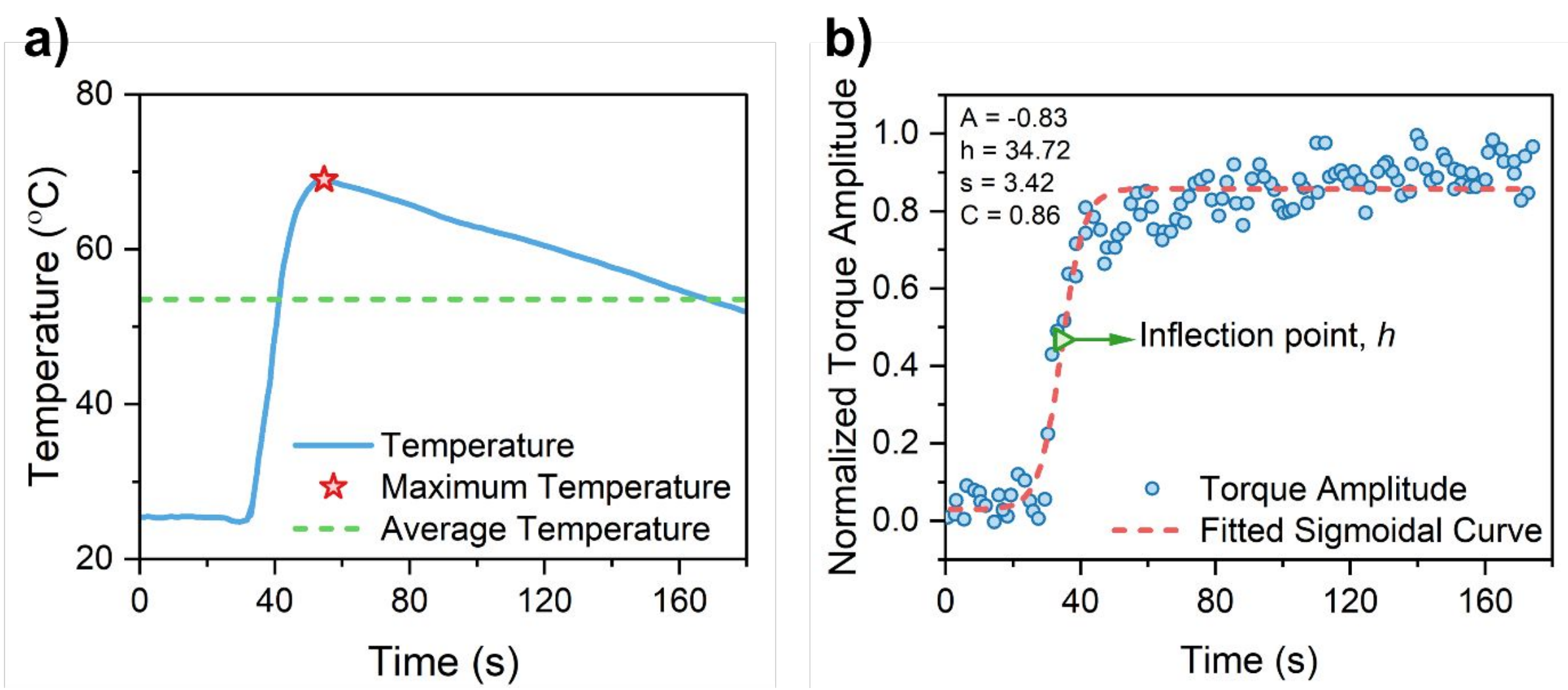

Figure S4. Example a) temperature curve and b) torque curve of a photopolymer ink during CTS test.

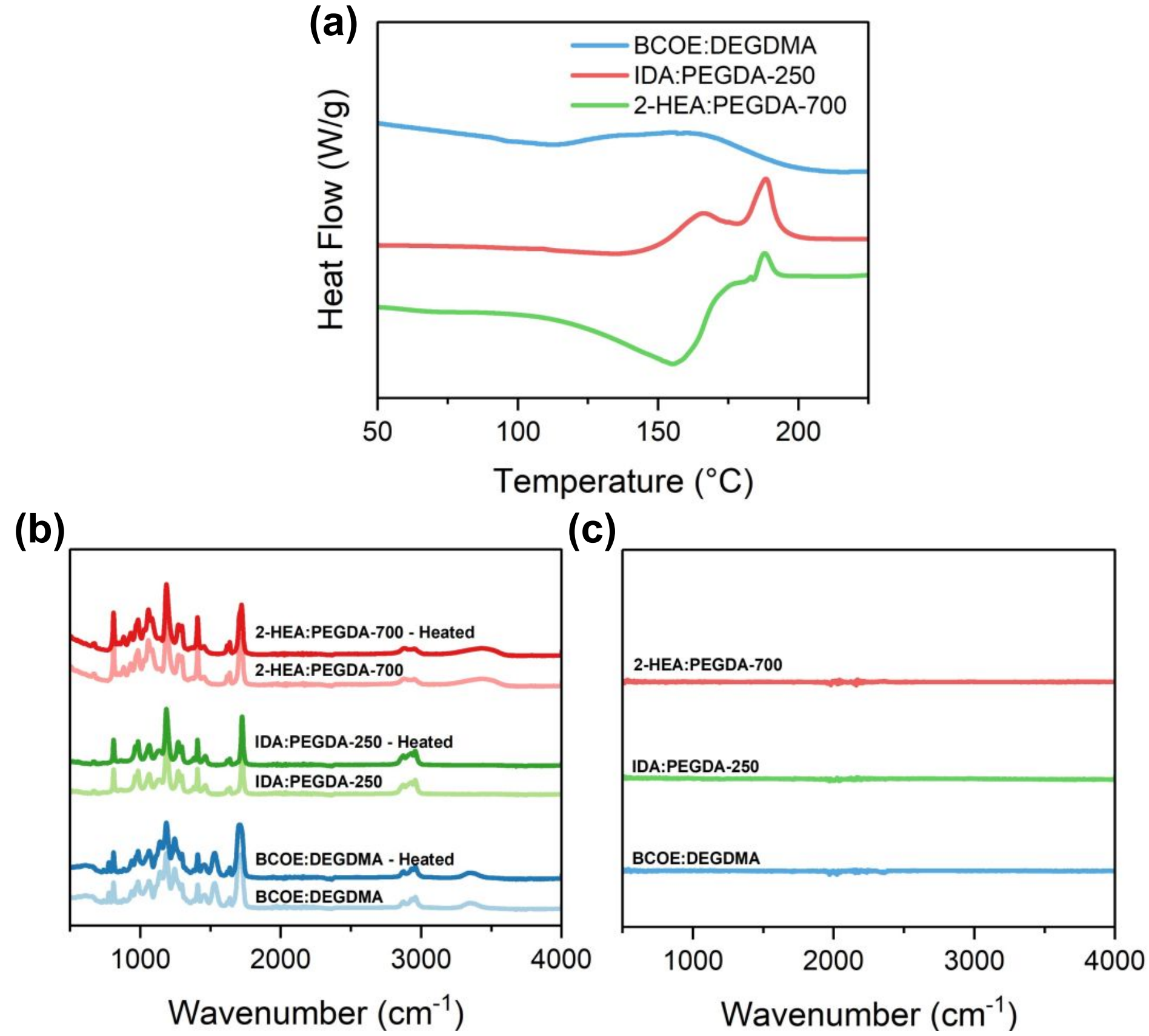

Figure S5. Evaluation of thermally induced polymerization in representative resin formulations. (a) DSC thermograms of BCOE:DEGDMA (87.4:12.6), IDA:PEGDA250 (75:25), and 2-HEA:PEGDA700 (80:20), showing no polymerization-related exotherm below 120 °C. (b) FTIR spectra of the three formulations before and after heating at 80 °C for 10 min. (c) Difference spectra between heated and unheated samples, showing no measurable change in the characteristic absorption bands after thermal treatment.

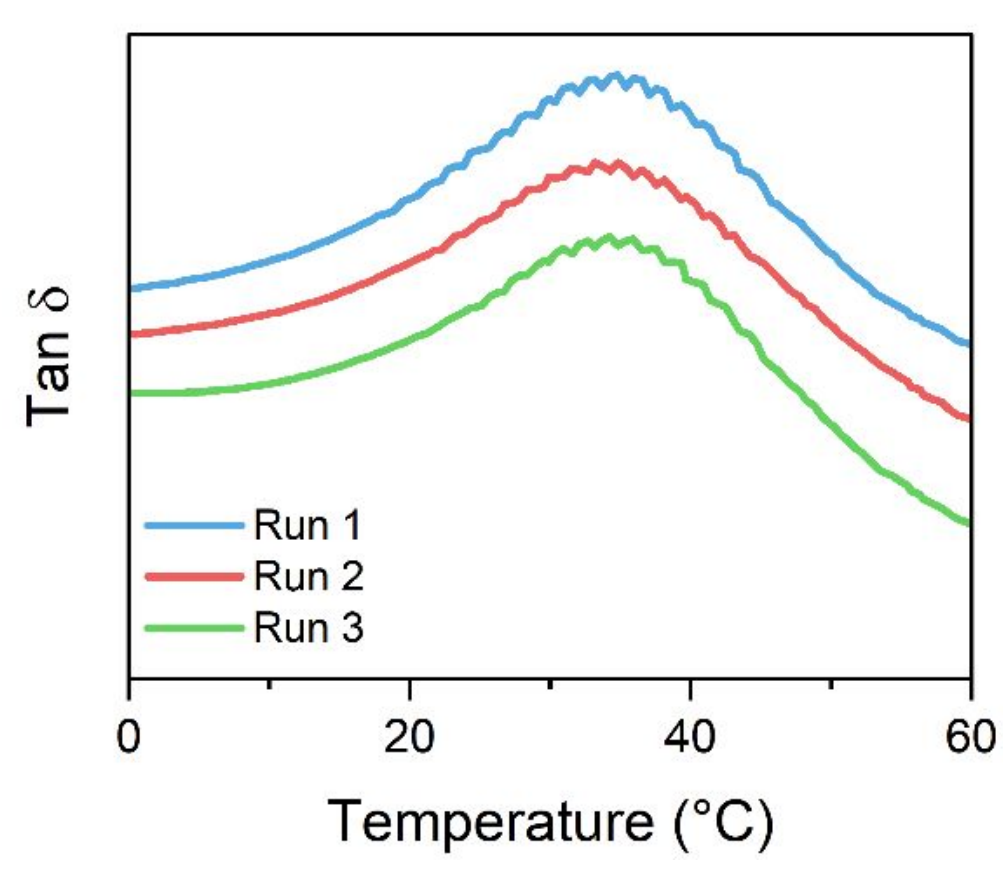

Figure S6. Comparison of Tan δ curves for one crosslinked polymer (PEGDA:GMA:AUD with weight ratio of 70:25:5) in different DMA runs to evaluate the accuracy of the DMA in Tg measurement.

2 Graph Neural Network Methodology

We adopt a Graph Attention Network (GAT)[1] to learn the relative importance of each monomer in a latent representation of the formulation, given the other monomers and the composition. For each node i, its updated representation at layer ℓ is given by **eq. S1**:

$$h_i^{(l)} = \|_{k=1}^{H} \sigma\left(\sum_{j\in N(i)} \alpha_{ij}^{(l,k)} W^{(l,k)} h_j^{(l-1)}\right) \tag{S2}$$

where $h_i^{(l-1)} \in R^{d_{in}}$ is the input feature of node i, $W^{(l,k)} \in R^{d_{out}\times d_{in}}$ is the learnable projection for head k, H is the number of attention heads, $\|$ denotes concatenation across heads, and $\sigma(\cdot)$ is a non-linear activation.

We use one GAT layer because the fully connected graph contains all the connections needed to represent the mixture. Node embeddings $\{h_i^{(L)}\}$, are aggregated into a graph-level embedding h_G via global mean pooling $h_G = \frac{1}{|V|}\sum_{i\in V} h_i^{(L)}$.

The pooled representation $h_G$is processed through a shared multilayer perceptron (MLP) with a one-hot encoding to extract task-independent features. From this shared embedding z, two separate task-specific heads are applied, with the property $\hat{y}_{property}$ as regression and realizability $\hat{y}_{realizability}$ as classification between 0 and 1. The first head predicts continuous molecular or reaction properties, while the second predicts a realizability score for feasibility classification.

The model is trained in a multi-task setting with a weighted loss:

$$\mathcal{L} = \lambda_{prop}\mathcal{L}_{MSE}\left(\hat{y}_{property}, y_{property}\right) + \lambda_{real}\mathcal{L}_{BCE}\left(\hat{y}_{realizability}, y_{realizability}\right) \tag{S3}$$

where $\mathcal{L}_{MSE}$ denotes mean squared error for regression, $\mathcal{L}_{BCE}$ denotes binary cross-entropy loss, and λ_{prop}, λ_{real} are hyperparameters controlling a weighting between tasks.

3 Hyperbolic Fitting for Glass Transition Temperature Details

The primary fitting function used to model the property-temperature relationship is a hyperbolic function:

$$\rho(T) = \rho_0 - \alpha(T - T_0) - b.H_0(T, T_0,c) \tag{S4}$$

where the hyperbolic component H_0 is defined as:

$$H_0(T, T_0, c) = \frac{1}{2}(T - T_0) + \sqrt{\frac{(T - T_0)^2}{4} + e^c} \tag{S5}$$

The transition probability function is given by:

$$P(T, T_0, c) = \frac{1}{2} + \frac{T - T_0}{2\sqrt{\frac{(T - T_0)^2}{4} + e^c}} \tag{S6}$$

The transition interval is calculated using a cutoff probability (P_{hat}):

$$\Delta T = \frac{e^{\frac{c}{2}}(2P_{hat} - 1)}{\sqrt{P_{hat}(1 - P_{hat})}} \tag{S7}$$

The transition interval bounds are then:

$$T_{low} = T_0 - \Delta T \tag{S8}$$

$$T_{high} = T_0 + \Delta T \tag{S9}$$

where $P_{hat} = 0.8$.

4 Gradient Optimization Details

4.1 Loss Functions

We implement three loss functions, tailored to different optimization scenarios:

Mean Squared Error (MSE): For exact target matching,

$$\mathcal{L}_{MSE}(f(x), t) = (f(x) - t)^2 \tag{S10}$$

Double Hinge Loss: For range-based optimization where $f(x) \in [t_{min}, t_{max}]$,

$$\mathcal{L}_{MSE}\big(f(x), [t_{min}, t_{max}]\big) = \begin{cases} 0, & t_{min} \leq f(x) \leq t_{max} \\ \min\big(|f(x) - t_{min}|, |f(x) - t_{max}|\big)^2, & otherwise \end{cases} \tag{S11}$$

Single Hinge Loss: For inequality constraints (maximize or minimize),

$$\mathcal{L}_{single}(f(x),t) = \max(0, 1 \mp f(x).t) \quad \text{(S12)}$$

4.2 Constraint Handling

To enforce simplex constraints, we use a softmax projection:

$$x_i^{proj} = \frac{\exp(x_i^{raw})}{\sum_{j=1}^{n} \exp(x_j^{raw})} \quad \text{(S13)}$$

4.3 Modified Adam Optimizer

We adopt a simplex-aware variant of Adam to ensure $\sum_{i=1}^{n} x_i = 1$. The update rules follow:

$$m_t = \beta_1 m_{t-1} + (1 - \beta_1) g_t \quad \text{(S14)}$$

$$v_t = \beta_2 v_{t-1} + (1 - \beta_2) g_t^2 \quad \text{(S15)}$$

$$\hat{m}_t = \frac{m_t}{1 - \beta_1^t} \quad \text{(S16)}$$

$$\hat{v}_t = \frac{v_t}{1 - \beta_2^t} \quad \text{(S17)}$$

$$\Delta x_i = \alpha \frac{\hat{m}_{t,i}}{\sqrt{\hat{v}_{t,i}} + \epsilon} + \frac{\eta_t}{t} N(0, 1) \quad \text{(S18)}$$

with the last component adjusted to preserve the constraint:

$$\Delta x_n = - \sum_{i=1}^{n-1} \Delta x_i \quad \text{(S19)}$$

A decaying noise term η_t/t provides exploratory behavior.

To account for predictive uncertainty, we employ Monte Carlo dropout. The GNN is kept in training mode, and multiple stochastic forward passes are averaged:

$$f_{mean}(x) = \frac{1}{N_{MC}} \sum_{i=1}^{N_{MC}} f_i(x) \quad \text{(S20)}$$

where N_{MC} (default: 10) is the number of samples. Gradients are computed with respect to f_{mean}. Sparse compositions are simplified by removing components with $x_i < 0.001$, along with their associated edges:

$$\varepsilon' = \{(i, j) \in \varepsilon : x_i > 0.001 \; and \; x_j > 0.001\} \quad \text{(S21)}$$

To mitigate local minima, we employ multi-start optimization. We run multiple starts with noise added to the sample from the Bayesian optimization.

Convergence is declared when

$$|\mathcal{L}_t - \mathcal{L}_{t-1}| < \tau\,, \qquad \tau = 10^{-6} \tag{S22}$$

To escape local minima, we apply a Dirichlet kick:

$$x_{new} = (1-\lambda)x_{current} + \lambda x_{Dirichlet} \tag{S23}$$

where $x_{\text{Dirichlet}}$ ~ Dirichlet($\kappa x_{\text{current}}$) with κ = 40 and λ = 0.25

4.4 Algorithm Summary

The complete optimization procedure is as follows:

1. Start from Bayesian Optimization chosen composition x_0.
2. For each iteration $t = 1, \ldots, T_{max}$:
   a. Enforce constraints: $x_t^{proj} = \prod(x_t)$
   b. Prune graph by removing zero-valued components.
   c. Forward pass through GNN (stochastic if enabled): $f_t = GNN(x_t^{proj})$
   d. Compute multi-objective loss: $\mathcal{L}_t = \sum_k \omega_k \mathcal{L}_k(f_{t,k}, t_k)$.
   e. Backpropagate to obtain gradients $\mathrm{g_t}$.
   f. Update composition with modified Adam.
   g. Check convergence: if converged for the first time, apply Dirichlet kick.
3. Terminate when convergence is achieved, $T_{max} = 1000$ is reached, or $\mathcal{L}_t < \tau$.
4. Return the best composition and its predicted properties.

5 Model for relationship between CTS and DLP 3D printing

The cure time of the ink from the CTS can provide information about the DLP curing time. The main differences between the two systems can be related by the Beer-Lambert Law.

$$\frac{dI}{dx} = -\mu I \tag{S24}$$

where I is the light intensity, μ is the "optical density" or absorption of the body, and x is the distance. As an approximation, we assume μ is a constant across the thickness. When integrated, it yields the equation below.

$$I = e^{-\mu x} \tag{S25}$$

From here, each system, DLP and CTS, must abide by the law, leading to

$$I_{DLP} = e^{-\mu h_p} \tag{S26}$$

$$I_{CTS} = e^{-\mu h_c} \tag{S27}$$

The typical layer height, h_p, for DLP is $50\mu m$, whereas the layer height for the ink in the glass container, h_c, is approximately 3.2mm, orders of magnitude difference. The absorption, μ, will be the same for identical inks in each system as it depends on the composition. The absorption may change between compositions, but the difference can be ignored for this rough estimation. Using the equations for each system, the degree of conversion, or degree of cure (DoC), can be assumed proportional to the light dose, It. The h_p is much smaller than h_c and can be assumed as zero.

$$\frac{I_{CTS}}{I_{DLP}} = \frac{e^{-\mu h_c}}{e^{-\mu h_p}} = e^{-\mu(h_c - h_p)} \approx e^{-\mu h_c} \tag{S28}$$

To reach the same DoC for both systems, the ratio between them can be found.

$$I_{CTS}t_{CTS} = I_{DLP}t_{DLP} \rightarrow \frac{t_{CTS}}{t_{DLP}} = \frac{I_{CTS}}{I_{DLP}} \tag{S29}$$

A rough estimation of the cure time is shown below.

$$t_{DLP} = t_{CTS}\frac{I_{CTS}}{I_{DLP}} \tag{S30}$$

To evaluate the values for the light intensities, a light meter was used for both systems. **Figure S7** shows the experimental setup for both the CTS and DLP systems. The light meter was placed on the exit of the light from the ink to find the resulting light intensity after decay,I. The DLP system has little to no decay because the layer is so thin. Therefore, the entrance and exit light intensities can be assumed to be the same, which is a value of 10.11 mW/cm^2. For the CTS system, the layer height is very thick and the light decays significantly. The resultant intensity is measured as 1.03 mW/cm^2. The quotient of these two numbers yields the $\frac{1}{10}$ fraction for the comparison between the cure times of each system, i.e.,

$$t_{DLP} \approx \frac{t_{CTS}}{10} \tag{S31}$$

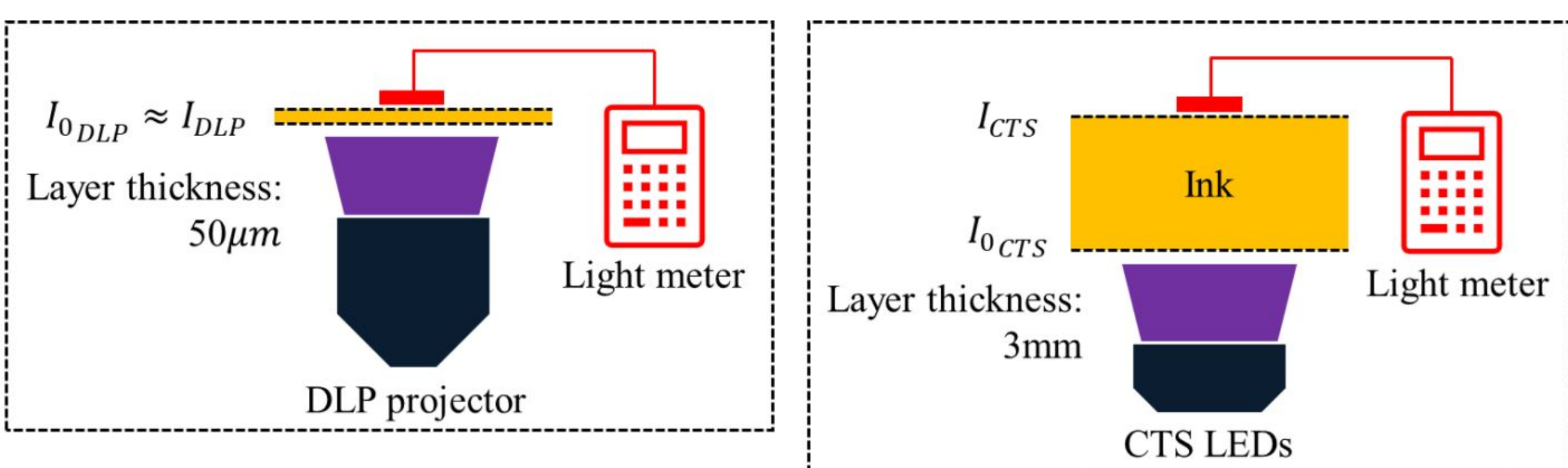

Figure S7. Experimental setup for the determination of light intensities between the DLP and CTS systems.

Based on this assumption, experiments were performed to estimate the curing time of the resin under DLP processing conditions. A 50 μm resin layer was placed between two glass slides separated by shims, and the slides were clamped together and exposed using the DLP projector. The cure time was identified as the exposure time at which the slides could no longer be readily separated. Following separation, the exposed layer was further examined to confirm the formation of a continuous, mechanically solid film with no visible uncured liquid resin. This criterion was used to determine the practical exposure time required to form a cured 50 μm layer under the DLP processing conditions.

6 Acrylate Monomers in *ThermosetDB*

Table S1.List of monomers with acronyms and SMILES

Name	Acronym	SMILES
Glycidyl Methacrylate	GMA	CC(=C)C(=O)OCC1CO1
Isobornyl acrylate	IBOA	[H][C@]12CC[C@@](C)([C@@H](C1)OC(=O)C=C)C2(C)C
Butyl Acrylate	BA	CCCCOC(=O)C=C
2-Hydroxyethyl acrylate	2-HEA	C=CC(=O)OCCO
2-Hydroxyethyl methacrylate	2-HEMA	CC(=C)C(=O)OCCO
Acrylic acid	AA	C=CC(=O)O
Isodecyl acrylate	IDA	CC(C)CCCCCCCOC(=O)C=C
2-Ethoxyethyl methacrylate	2-EEMA	CCOCCOC(=O)C(C)=C
tert-Butyl Acrylate	t-BA	CC(C)(C)OC(=O)C=C
Benzyl methacrylate	BMA	CC(=C)C(=O)OCc1ccccc1
2-[[(Butylamino)carbonyl]oxy]ethyl acrylate	BCOE	CCCCNC(=O)OCCOC(=O)C=C
Poly-ethylene Glycol Diacrylate	PEGDA	C=CC(=O)OCCOCCOCCOC(=O)C=C
Bisphenol A ethoxylate diacrylate	BPAEDA-500	C=CC(=O)OCCOCC(C)(c1ccc(O)cc1)c2ccc(O)cc2OCCOCCOC(=O)C=C
Bisphenol A ethoxylate diacrylate	BPAEDA-700	C=CC(=O)OCCOCCOCCOCCOCC(C)(c1ccc(O)cc1)c2ccc(O)cc2OCCOCCOCCOCCOC(=O)C=C
Trimethylolpropane ethoxylate triacrylate	ETPTA	CCC(COCCOC(=O)C=C)(COCCOC(=O)C=C)COCCOC(=O)C=C
Ethylene glycol phenyl ether acrylate	PA	C=CC(=O)OCCOc1ccccc1
2-(Diethylamino)ethyl methacrylate	DEAEMA	CCN(CC)CCOC(=O)C(C)=C
Poly-ethylene Glycol Diacrylate 575	PEG-575	C=CC(=O)OCCOCCOCCOCCOCCOCCOCCOCCOCCOC(=O)C=C
Poly-ethylene Glycol Diacrylate 700	PEG-700	C=CC(=O)OCCOCCOCCOCCOCCOCCOCCOCCOCCOCCOCCOCCOC(=O)C=C
Poly-ethylene Glycol Diacrylate 300	PEGDMA-300	C=CC(=O)OCCOCCOCCOCCOC(=O)C=C
Di(ethylene glycol) dimethacrylate	DEGDMA	CC(=C)C(=O)OCCOCCOC(=O)C(C)=C
Aliphatic Urethane Diacrylate	AUD	C=CC(=O)OCOC(=O)NCNC(=O)OCOC(=O)C=C
Poly(ethylene glycol) dimethacrylate	PEGDMA	C=C(C)C(=O)OCCOCCOCCOCCOCCOCCOCCOCCOC(=O)C(=C)C
Methyl acrylate	MA	COC(=O)C(C)=C
Methyl methacrylate	MMA	COC(=O)C(C)=C
tert-Butyl methacrylate	tBMA	CC(=C)C(=O)OC(C)(C)C
Isobornyl methacrylate	IMA	CC(=C)C(=O)OC1C[C@H]2CC[C@]1(C)C2(C)C
2-Ethylhexyl methacrylate	2EHM	CCCCCC(CC)COC(=O)C(C)=C
Ethylene glycol phenyl ether methacrylate	EGPEM	CC(=C)C(=O)OCCOc1ccccc1
Poly(propylene glycol) acrylate	PPGA	C=CC(=O)OCC(C)OCC(C)OCC(C)OCC(C)OCC(C)OCC(C)OCC(C)O
Poly(ethylene glycol) phenyl ether acrylate (Mn 236)	PEGPEA236	C=CC(=O)OCCOCCOc1ccccc1
Poly(ethylene glycol) phenyl ether acrylate (Mn 280)	PEGPEA280	C=CC(=O)OCCOCCOCCOc1ccccc1
Poly(ethylene glycol) phenyl ether acrylate (Mn 324)	PEGPEA324	C=CC(=O)OCCOCCOCCOCCOc1ccccc1
Benzyl acrylate	BZA	O(Cc1ccccc1)C(=O)C=C
Bisphenol A ethoxylate diacrylate Mn 688	BPA688	C=CC(=O)OCCOCCOCCOc1ccc(cc1)C(C)(C)c2ccc(cc2)OCCOCCOCCOCCOC(=O)C=C
Bisphenol A ethoxylate diacrylate Mn 468	BPA468	C=CC(=O)OCCOCCOc1ccc(cc1)C(C)(C)c2ccc(cc2)OCCOC(=O)C=C
1,6-Hexanediol diacrylate	HEXDA	C=CC(=O)OCCCCCCOC(=O)C=C
Trimethylolpropane ethoxylate triacrylate Mn 604	TETA604	C=CC(=O)OCCOCCOCCOCC(CC)(COCCOCCOC(=O)C=C)COCCOCCOC(=O)C=C
Trimethylolpropane ethoxylate triacrylate Mn 912	TETA912	C=CC(=O)OCCOCCOCCOCCOCCOCC(CC)(COCCOCCOCCOCCOCCOC(=O)C=C)COCCOCCOCCOC COC(=O)C=C
Glycerol propoxylate triacrylate	GPTA	C=CC(=O)OCCCOCC(COCCCOC(=O)C=C)OCCCOC(=O)C=C

Di(trimethylolpropane) tetraacrylate	DTTA	CCC(COCC(CC)(COC(=O)C=C)COC(=O)C=C)(COC(=O)C=C)COC(=O)C=C
Dipentaerythritol penta/hexaacrylate	DPPHA	C=CC(=O)OCC(CO)(COCC(COC(=O)C=C)(COC(=O)C=C)COC(=O)C=C)COC(=O)C=C
Poly-ethylene Glycol Diacrylate 700	PEGDMA750	C=C(C)C(=O)OCCOCCOCCOCCOCCOCCOCCOCCOCCOCCOCCOCCOCCOC(=O)C(=C)C
Bisphenol A ethoxylate dimethacrylate Mn 1700	BPA1700	C=C(C)C(=O)OCCOCCOCCOCCOCCOCCOCCOCCOCCOCCOCCOCCOCCOCCOCCOc1ccc(cc1)C(C)(C)c2ccc(cc2)OCCOCCOCCOCCOCCOCCOCCOCCOCCOCCOCCOCCOCCOCCOCCOC(=O)C(C)=C
Bisphenol A ethoxylate dimethacrylate Mn 540	BPA540	C=C(C)C(=O)OCCOCCOc1ccc(cc1)C(C)(C)c2ccc(cc2)OCCOCCOC(=O)C(C)=C
Octyl Methacrylate	OctMA	CCCCCCCCOC(=O)C(=C)C
Heptyl Methacrylate	HepMA	CCCCCCCCOC(=O)C(=C)C
Diethylene glycol diacrylate	PEGDA-2	C=CC(=O)OCCOCCOC(=O)C=C
Pentaerythritol Tetracrylate	PETA	C=CC(=O)OCC(COC(=O)C=C)(COC(=O)C=C)COC(=O)C=C
2,2-bis[4(2,3-hydroxypropoxy)phenyl]propane	Bis-HPPP	CC(C1=CC=C(OCC(O)COC(C(C)=C)=O)C=C1)(C2=CC=C(OCC(O)COC(C(C)=C)=O)C=C2)C
Triethylene glycol dimethacrylate	TEGDMA	C=C(C)C(=O)OCCOCCOCCOC(=O)C(=C)C
Poly-ethylene Glycol Diacrylate 600	PEGDMA-600	C=C(C)C(=O)OCCOCCOCCOCCOCCOCCOCCOCCOCCOCCOC(=O)C(=C)C
Neopentyl glycol propoxylate diacrylate	NGPDA	C=CC(=O)OCCCOCC(C)(C)COCCCOC(=O)C=C
Trimethylolpropane propoxylate triacrylate	TPTA	C=CC(=O)OCCCOCCCOCC(CC)(COCCCOCCCOC(=O)C=C)COCCCOCCCOC(=O)C=C
Urethane Dimethacrylate	UDMA	C=CC(=O)OCCOCCOC(=O)C=CCC(CCNC(=O)OCCOC(=O)C(C)=C)CC(C)(C)CNC(=O)OCCOC(=O)C(C)=C

7 Exploration Active Learning

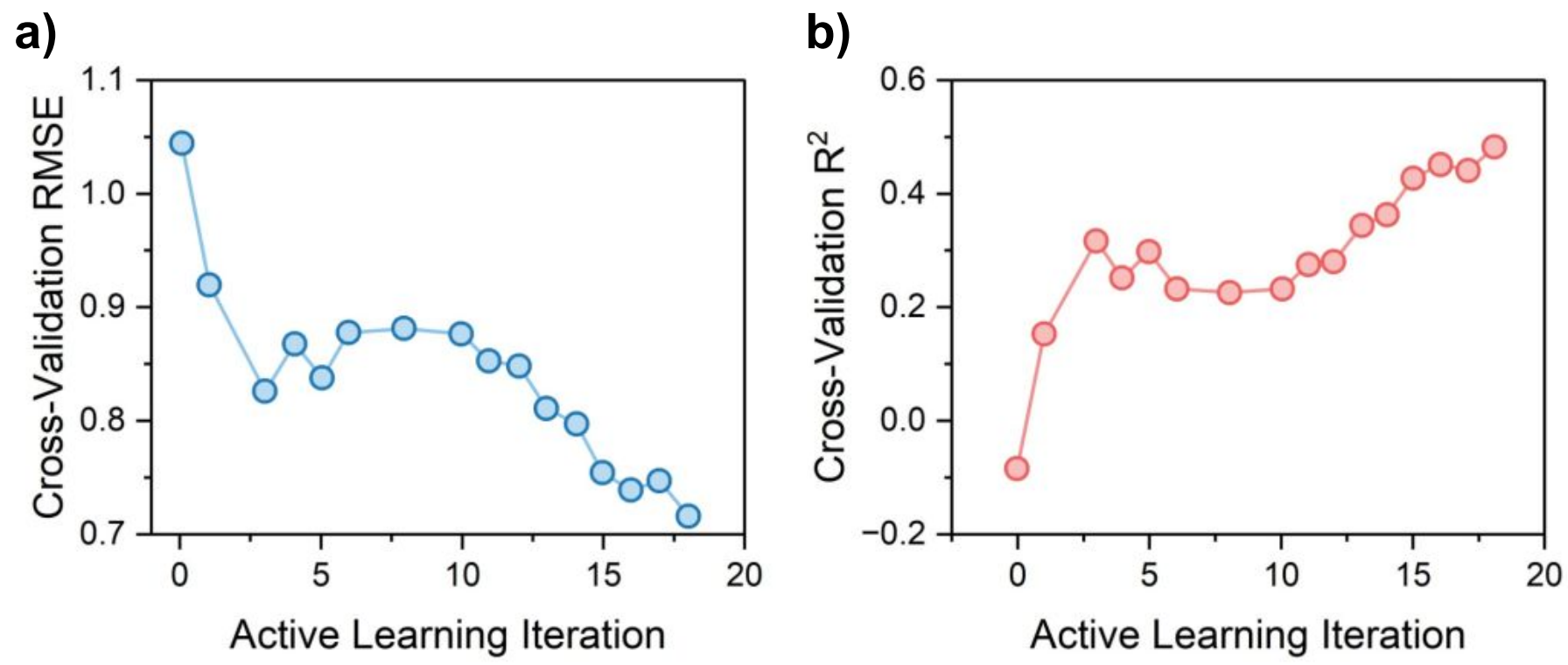

Figure S8. Results of the normalized RMSE and r^2 over several iterations of exploration-based acquisition across the acrylate formulation space.

8 References

(1) Velickovic, P.; Cucurull, G.; Casanova, A.; Romero, A.; Lio, P.; Bengio, Y. Graph Attention Networks. International Conference on Learning Representations. 2018.
